# Supplementary material for: Small reorganization energy acceptors enable low energy losses in non-fullerene organic solar cells
Source: Nat Commun. 2022 Jun 7;13:3256. doi: 10.1038/s41467-022-30927-y (PMC9174259; doi:10.1038/s41467-022-30927-y)
Supplement: Supplementary file 1 — Supplementary Information [file 41467_2022_30927_MOESM1_ESM.pdf]

Supplementary Information

Small reorganization energy acceptors enable low energy losses in non-fullerene organic solar cells

Shi, Chang et al.

Supplementary Information

Small reorganization energy acceptors enable low energy losses in non-fullerene organic solar cells

Yanan Shi [1,2,‡], Yilin Chang [1,2,‡], Kun Lu [1,2,*], Zhihao Chen [3], Jianqi Zhang [1], Yangjun Yan [1] , Dingding Qiu [1,2], Yanan Liu [1], Muhammad Abdullah Adil [1], Wei Ma [4], Xiaotao Hao[3,*], Lingyun Zhu [1,*], and Zhixiang Wei [1,2,*]

[1] Chinese Academy of Sciences (CAS) key laboratory of nanosystem and hierarchical fabrication, National Center for Nanoscience and Technology, Beijing 100190, China

Email: *lvk@nanoctr.cn*; *zhuly@nanoctr.cn*; *weizx@nanoctr.cn*

[2] University of Chinese Academy of Sciences, Beijing 100049, China

[3] School of Physics, State Key Laboratory of Crystal Materials, Shandong University, Jinan, Shandong 250100, China

Email: *haoxt@sdu.edu.cn*

[4] State Key Laboratory for Mechanical Behavior of Materials, Xi'an Jiaotong University, Xi'an 710049, China

[‡] These authors contributed equally: Yanan Shi, Yilin Chang

Supplementary Methods

Molecular structure characterization

^{1}H NMR was obtained on a Bruker DMX-400 NMR Spectrometer (operating at 400 MHz, using $CDCl_3$ as solvent using tetramethylsilane as internal standard). Mass spectra were measured by Bruker microflex MALDI-TOF mass spectrometer.

Single-crystal growth

Single crystals of Qx-1 and Qx-2 were grown by the liquid diffusion method at room temperature. An appropriate amount of methanol is transferred to a concentrated chloroform solution, which will form crystals over time. Single crystal diffraction was collected at low temperatures protected by liquid nitrogen in accordance with standard procedures for reducing X-ray radiation damage through use single-crystal X-ray diffractometer (model is XtaLAB PRO 007HF(Mo), manufactured by Rigaku). The X-ray crystallographic coordinates for structures reported of Qx-1 and Qx-2 have been deposited at the Cambridge Crystallographic Data Centre (CCDC), under deposition numbers 2120380–2120381.

Molecular properties characterization

The UV-vis absorption was measured by Perkin Elmer Lambda 950 spectrophotometer.

The electrochemical cyclic voltammetry (CV) was conducted an electrochemical workstation (VMP3 Biologic, France) with a Pt disk coated with blend film, a Pt plate, and an Ag^+/Ag electrode acting as the working, counter, and reference electrodes, respectively, in a 0.1mol/L tetrabutylammonium phosphorus hexafluoride (Bu_4NPF_6) acetonitrile solution. The experiments were calibrated with the standard ferrocene/ferrocenium (Fe) redox system and assumption that the energy level of Fe is 4.8 eV below the vacuum, through using the formula $E_{HOMO/LUMO} = -e(E_{ox/red} - E^{1/2}(Fe/Fe^+) + 4.8)$ (eV) through the onset oxidation (E_{ox}) and reduction (E_{red}) potentials.[4, 5]

Reorganization energy calculations

In the calculations, the alkyl chains in the acceptor molecules were replaced by ethyl groups for simplification. The geometry optimizations and frequency analyses of all the molecules were performed by using Density Functional theory (DFT) for the S_0 and anion states and using time-dependent DFT (TDDFT) for the S_1 state. Here, the long-range corrected (LRC) functional ωB97XD and 6-31G (d, p) basis set were employed.

Moreover, to obtain a more reliable description of the electronic properties, the range separation parameter (ω) of the LRC functional was optimized by changing ω to minimize the value of $J(\omega)$,[6-8]

$$J(\omega)= J_{\mathrm{IP}}(\omega) + J_{\mathrm{EA}}(\omega) \tag{2}$$

$$J_{\mathrm{IP}}(\omega)=\left|E_{+}(\omega) - E_{0}(\omega)+\varepsilon_{0}^{\mathrm{H}}(\omega)\right| \tag{3}$$

$$J_{\mathrm{EA}}(\omega)=\left|E_{0}(\omega)-E_{-}(\omega)+\varepsilon_{-}^{\mathrm{H}}(\omega)\right| \tag{4}$$

where $\varepsilon_{0}^{\mathrm{H}}$ and $\varepsilon_{-}^{\mathrm{H}}$ are the HOMO energies in the neutral and anionic states, respectively. The optimal ω value is 0.096, 0.097 and 0.105 for the Qx-1, Qx-2 and Y6 molecules, respectively.

The vibrational frequencies of the optimized geometries for the S_0, S_1, and anion states are all positive, indicating that all the obtained geometries are stable. The results of vibration calculations were used to calculate the reorganization energies for the electronic transitions among the S_0, S_1, and anion states with the MOMAP code.[9] All the calculations for the electronic structure properties were carried out with the Gaussian 09 program.[10]

Molecular Packing Simulations

The atomistic MD simulations were performed using the Gromacs 4.6.7 software package.[11] The atom types and intra- and inter-molecular interaction parameters of Qx-1 and Qx-2 were built from the general AMBER force field (GAFF) with the RESP charges. The torsion potential between the central

backbone and the terminal acceptor group was reparametrized according to DFT calculations. The thin-film molecular packing structures of Qx-1 and Qx-2 were obtained according to the following procedure: (i) constructing a 25×25×25 nm^3 box containing 400 Qx-1 or Qx-2 molecules with different conformations and 50000 chloroform molecules by randomly placing Qx-1 or Qx-2 molecules into the solvent; (ii) 2 ns simulation under high pressure (100 bar) and then 20 ns simulation under normal pressure (1 bar) at room temperature (300 K) to make the Qx-1 or Qx-2 molecules and solvent molecules disperse homogeneously; (iii) randomly removing 100 solvent molecules from the solution every 200 ps to simulate the solvent evaporation process at room temperature and normal pressure with the compressibility of 1.0×10^{-5} bar^{-1}; (iv) after removing all of the solvent molecules, 60 ns equilibration was carried out at 300 K and 1 bar. The velocity rescaling thermostat and the Berendsen barostat under the NPT ensemble were applied to control the temperature and pressure, respectively. For the final 30 ns of equilibration, the Nose–Hoover thermostat, and the Parrinello–Rahman barostat were used to obtain better equilibrium conformations.

Device fabrication and measurement.

All the PSC devices were fabricated by using an conventional structure of Indium tin oxide(ITO)/poly(3,4-ethylenedioxythiophene:poly(styrenesulfonate)(PEDOT:PSS) /PM6:SMA/poly[(9,9-bis(3'-((**N**,**N**-dimethyl)-**N**-ethylammonium)propyl)-2,7 fluorene)-**alt**-5,5′-bis(2,2′-thiophene)-2,6-naphthalene-1,4,5,8-tetracaboxylic-**N**,**N**′-di(2ethylhexyl)imide]dibromide(PNDIT-F3N-Br)/Ag, where PNDIT-F3N-Br and PEDOT:PSS were respectively used as electron-transport and hole-transport interlayer. The ITO glass substrates were cleaned sequentially under sonication with detergent, deionized water , ethyl alcohol and isopropyl alcohol. After oxygen plasma cleaning for 15 min, the PEDOT:PSS layer was deposited by spin-

coating under 3500 rpm for 30 s on top of the ITO substrate with thermal annealed for 15 minutes at 150 °C. The blended solution was prepared by mixing PM6 and acceptors into chloroform (CF) with the addition of a small amount of chloronaphthalene (0.6 %, v/v). The mix solution was stirred at 50 °C in chloroform for 1.5 h until they dissolved. The optimal device conditions of Qx-1 and Qx-2 were prepared by mixing donor and acceptors in a 1:1.5 and1:1.3 weight ratio, respectively, into chloroform (CF) with the addition of a small amount of 1-chloronaphthalene (CN) (0.6 %, v/v) and under thermal annealing at 100 °C and 110°C for 10 min, respectively. The ETLs dissolved in methanol at a concentration of 0.5 mg/ml were spun onto the blend layer at 3000 rpm for 30 s. Finally, 160 nm-thick Ag was deposited onto the ETLs layer under vacuum at a pressure of 3×10^{-6}mbar. Except for the fabrication of PEDOT:PSS layer, the other processes were all carried out in a nitrogen-filled glovebox. A solar simulator was used for *J–V* curves measurement under AM 1.5 G (100mWcm^{-2}). Newport Oriel PN 91150 V Si-based solar cell was applied for light intensity calibration. *J–V* measurement signals were recorded by a Keithley 2400 source-measure unit. The device area of each cell was approximately 4mm^2. Oriel Newport system (Model 66902) equipped with a standard Si diode was used for EQEs test in air condition.

The J_{SC} and V_{OC} under different light intensity. In organic solar cell devices, the relationship between J_{SC} and P_{light} can be expressed by the formula $J_{SC} \propto (P_{light})^{\alpha}$, where α is the power exponent. When the value of α in the formula approaches 1, the bimolecular recombination in the device can be ignored.[12] the V_{OC} and ln(P_{light}) can be fitted to a straight line, and the slope of the fitted line should be $k\mathrm{T}/q$ (where k is Boltzmann's constant, T is the Kelvin temperature, and q is the elementary charge).[13]

The dependence of the photocurrent density (J_{ph}) on the effective voltage (V_{eff}) was also tested to analyze exciton dissociation and charge collection. J_{ph} was obtained from $J_{ph} = J_L - J_D$ (J_L and J_D are

the current densities under illumination and dark conditions), and V_{eff} was calculated from $V_{eff} = V_0 - V$ (V_0 is the voltage when $J_L = J_D$ and V is the applied voltage).[14] Under short-circuit conditions, the exciton dissociation ($P_{diss} = J_{ph}/J_{sat}$, where J_{sat} represents the saturation photocurrent density).

Mobility measurements are the same for solar cell devices. The electron mobility was acquired with the device structure of Al/active layer or neat acceptors/PNDIT-F3N-Br/Al, the hole mobility was obtained by preparing the structure of ITO/PEDOT:PSS/active layer/MoOx/Ag. The current density-voltage (J-V) curves in the range of 0-5 V were obtained by a Keithley 2420 Source-Measure Unit in the dark. Electron and hole motilities were measured with electron- and hole-only devices by using the SCLC model[15], which can be calculated by fitting the results in the equation:

$$J = \frac{9}{8}\varepsilon_r\varepsilon_0\mu_0\frac{(V-V_{bi})^2}{L^3}\exp\left(\beta\sqrt{\frac{V-V_{bi}}{L}}\right) \tag{5}$$

where J is the current density, L is the film thickness of active layer, μ is the hole mobility, ε_r is the relative dielectric constant of the transport medium. It is assumed to be 3, which is typical for conjugated organic materials. ε_0 is the permittivity of free space, V is the internal voltage in the device and $V = V_{appl} - V_r - V_{bi}$, where V_{appl} is the applied voltage to the device, V_r is the voltage drop due to contact resistance and series resistance across the electrodes, and V_{bi} is the built-in voltage due to the relative work function difference of the two electrodes.

Morphology characterization

All morphologies were characterized by films prepared under the same conditions. Transmission electron microscopy (TEM) images were acquired on Tecnai G2 F20 U-TWIN TEM instrument. The atomic force microscopy (AFM) characterization was performed by Bruker Multimode 8 in ScanAsyst Mode in air. Grazing incidence wide angle x-ray scattering (GIWAXs) measurement was conducted at the beamline of 7.3.3 at the Advanced Light Source (ALS). Samples were prepared on Si/PEDOT: PSS

substrates. Resonant Soft X-ray Scattering (RSoXS): RSoXS transmission measurements were performed at beamline 11.0.1.2[2] at the Advanced Light Source (ALS). (Samples for were prepared on a PSS modified Si substrate under the same conditions as those used for device fabrication, and then transferred by floating in the water to a 1.5 mm × 1.5 mm, 100 nm thick Si_3N_4 membrane supported by a 5 mm × 5 mm, 200 μm thick Si frame (Norcada Inc.). 2-D scattering patterns were collected on an in-vacuum CCD camera (Princeton Instrument PI-MTE). The sample detector distance was calibrated from diffraction peaks of a triblock copolymer poly(isoprene-b-styrene-b-2-vinyl pyridine), which has a known spacing of 391 Å. The beam size at the sample is approximately 100 μm by 200 μm.

Exciton and Charge Dynamics

The excitation and emission spectra, the time-resolution photoluminescence (TRPL) spectrum, and absolute quantum yield (QY) test by transient/steady-state fluorescence spectrometer, manufactured by Edinburgh Instruments, model number is FLS1000. The photoluminescence (PL) spectrum of film and solution use a xenon lamp to excite light source, use 750nm pump for film excitation, and use 600nm pump for solution excitation. Absolute quantum yield using an integrating sphere and a blank quartz plate as a reference to measure the absolute quantum yield.

The exciton annihilation method was used to measure exciton diffusion constants. This method employs ultrafast TA spectroscopy to measure exciton lifetimes as a function of excitation density. Annihilation occurs when excitons collide and exchange energy, thus the diffusion constant is readily obtained from the bimolecular rate constant for a series of known excitation densities. A series of excitation fluences dependent decays are globally fit to a rate equation accounting for bimolecular (exciton annihilation) and monomolecular decay pathways, assuming that annihilation destroys both

excitons;

$$\frac{\mathrm{dn}(t)}{\mathrm{d}t} = -k\mathrm{n}(t) - \frac{1}{2}\gamma \mathrm{n}^2(t) \tag{6}$$

$$\mathrm{n(t)} = \frac{\mathrm{n}(0)\mathrm{e}^{(-kt)}}{1+\frac{\gamma}{2k}\mathrm{n}(0)[1\text{-}\mathrm{e}^{(-kt)}]} \tag{7}$$

where n(t) is the singlet exciton density as a function of time after the laser excitation, k is the monomolecular decay rate and γ is the singlet-singlet bimolecular exciton annihilation rate.

The bimolecular rate constant is then used to determine the 3D exciton diffusion coefficiet;

$$D = \frac{\gamma}{8\pi R} \tag{8}$$

where *D* is the diffusion constant and *R* is the effective interaction or annihilation radius of singlet excitons which is the separation at which the annihilation occurs.[16]

TA measurements were performed on an Ultrafast Helios pump-probe system in collaboration with a regenerative amplified laser system from Coherent. An 800 nm pulse with a repetition rate of 1k Hz, a length of 100 fs, and an energy of 7 mJ per pluse, was generated by an Ti:sapphire amplifier (Astrella, Coherent). Then the 800 nm pulse was separated into two parts by a beam splitter. One part was coupled into an optical parametric amplifier (TOPAS, Coherent) to generate the pump pulses at various wavelength. The other part was focused onto a sapphire plate and a YAG plate to generate white light supercontinuum as the probe beams with spectra covering 420-800nm and 750-1600 nm, respectively. The time delay between pump and probe was controlled by a motorized optical delay line with a maximum delay time of 8 ns. The pump pulse is chopped by a mechanical chopper with 500 Hz and then focused on to the mounted sample with probe beams. The probe beam was collimated and focused into a fiber-coupled multichannel spectrometer with CCD sensor. The energy of pump pulse was measured and calibrated by a power meter (PM400, Thorlabs). All samples used for PL and TA measurements were obtained by spin-coating the neat and blend solutions on the quartz substrates.

Pump wavelength was set to 400nm and 800nm to selectively excite the donor and acceptors in blend films.

Energy loss

Highly sensitive EQE was measured using an integrated system (PECT-600, Enlitech), where the photocurrent was amplified and modulated by a lock-in instrument. EQE_{EL} measurements were performed by applying external voltage/current sources through the devices (ELCT-3010, Enlitech). EQE_{EL} measurements were performed for all devices according to the optimal device preparation conditions.

Supplementary Discussion

The chemical name of materials:

Qx-1: 2, 2'-((2Z,2'Z)-((13,14-bis(2-butyloctyl)-6,7-diphenyl-3,10-diundecyl-13,14 dihydrothieno [2",3":4',5']thieno[2',3':4,5]pyrrolo[3,2 f]thieno[2",3":4',5']thieno[2',3':4,5]pyrrolo[2,3-h]quinoxaline-2,11-diyl)bis(methaneylylidene))bis(5,6-difluoro-3-oxo-2,3-dihydro-1H-indene-2,1-diylidene))dimalononitrile.

Qx-2: 2,2'-((2Z,2'Z)-((10,11-bis(2-butyloctyl)-7,14-diundecyl-10,11 dihydrodibenzo[a,c]thieno [2",3":4',5']thieno[2',3':4,5]pyrrolo[3,2-h]thieno[2",3":4',5']thieno[2',3':4,5]pyrrolo[2,3-j]phenazine-8,13-diyl)bis(methaneylylidene))bis(5,6-difluoro-3-oxo-2,3-dihydro-1H-indene-2,1-diylidene)) dimalononitrile.

BTP-C4C6: 12,13-bis(2-butyloctyl)-3,9-diundecyl-12,13-dihydro-[1,2,5]thiadiazolo[3,4-e]thieno [2",3":4',5']thieno[2',3':4,5]pyrrolo[3,2-g]thieno[2',3':4,5]thieno[3,2-b]indole.

Qx-1 core: 13,14-bis(2-butyloctyl)-6,7-diphenyl-3,10-diundecyl-13,14-dihydrothieno [2",3":4',5'] thieno[2',3':4,5]pyrrolo[3,2-f]thieno[2",3":4',5']thieno[2',3':4,5]pyrrolo[2,3-h]quinoxaline.

Qx-2 core: 10,11-bis(2-butyloctyl)-7,14-diundecyl-10,11-dihydrodibenzo[a,c]thieno[2",3":4',5'] thieno[2',3':4,5]pyrrolo[3,2-h]thieno[2",3":4',5']thieno[2',3':4,5]pyrrolo[2,3-j]phenazine.

Qx-1-CHO: 13,14-bis(2-butyloctyl)-6,7-diphenyl-3,10-diundecyl-13,14-dihydrothieno [2",3":4',5']thieno[2',3':4,5]pyrrolo[3,2-f]thieno[2",3":4',5']thieno[2',3':4,5]pyrrolo[2,3-h]quinoxaline-2,11-dicarbaldehyde.

Qx-2-CHO: 10,11-bis(2-butyloctyl)-7,14-diundecyl-10,11-dihydrodibenzo[a,c]thieno[2",3":4',5'] thieno[2',3':4,5]pyrrolo[3,2-h]thieno[2",3":4',5']thieno[2',3':4,5]pyrrolo[2,3-j]phenazine-8,13-dicarbaldehyde.

Compound 5: 2-(5,6-difluoro-3-oxo-2,3-dihydro-1H-inden-1-ylidene)malononitrile.

The synthetic route of acceptors Qx-1 and Qx-2 is illustrated in Supplementary Figure 2. After compound 1 (BTP-C4C6) is reduced, compound 3-1 (Qx-1 core) or compound 3-2 (Qx-2 core) is obtained by Schiff base reaction with compound 2-1 (benzil) or compound 2-2 (phenanthrene-9,10-dione).[1, 2] Compound 3-1 (Qx-1 core) or compound 3-2 (Qx-2 core) with the newly prepared Vilsmeier reagent (DMF and $POCl_3$) in 1,2-dichloroethane produced compound 4-1 (Qx-1-CHO) or compound 4-2 (Qx-2-CHO), respectively.[3] The target molecules Qx-1 and Qx-2 were synthesized by a Knoevenagel reaction between compound 4-1 or compound 4-2 (Qx-2-CHO) and electron-withdrawing end-groups in a high yield over 80%. Compound 1 and compound 5 were purchased from Hyper Chemical Company. Compound 2-1 and compound 2-2 and were purchased from Bide Pharmatech Ltd. Unless otherwise specified, all medicines are purchased from commercial sources and used directly without further purification. All the reagents and solvents used in the experiment are of analytical purity and are used directly without purification.

Compound 3-1 (Qx-1 core): To a solution of compound 1(1.00 g, 0.923mmol) in acetic acid (50 mL) was added zinc powder (2.40 g, 36.91mmol) in one portion. Then the mixture solution was heated to 90°C for 5 h. After the solution was cooled at room temperature, the solid was removed by filtration. Transfer the filtrate to a three-tip flask containing compound 2-1 (776mg, 3.69mmol), then the mixture solution was heated to 110°C for 20 h. After cooling to room temperature, washed with saturated salt water and methylene chloride. The solvent was removed under reduced pressure. The crude product was subsequently purified by column chromatography on silica gel to afford compound 3-1 as yellow oily solid (280mg, 25% yield). ^{1}H NMR (400 MHz, CDCl3) δ 7.74 (d, J = 6.4 Hz, 4H), 7.38 – 7.30 (m, 6H), 6.93 (s, 2H), 4.59 (d, J = 7.9 Hz, 4H), 2.78 (t, J = 7.6 Hz, 4H), 1.80 (dt, J = 14.6, 7.4 Hz, 4H),

1.19 (d, J = 5.4 Hz, 44H), 0.83 – 0.77 (m, 22H), 0.60 (tt, J = 7.3, 5.4 Hz, 18H).

Compound 3-2 (Qx-2 core): was synthesized by similar procedure as compound 3-1 between compound 1 and compound 2-2. The final product were obtained as yellow solid (260mg, 23% yield). ^{1}H NMR (400 MHz, CDCl3) δ 9.78 (d, J = 7.4 Hz, 2H), 8.73 (d, J = 8.1 Hz, 2H), 7.91 (dd, J = 30.3, 7.5 Hz, 4H), 7.07 (s, 2H), 4.73 (d, J = 7.7 Hz, 4H), 2.97 (s, 4H), 2.00 (d, J = 14.7 Hz, 4H), 1.51 (d, J = 45.8 Hz, 26H), 1.29 (s, 26H), 0.96 (s, 14H), 0.89 (t, J = 6.1 Hz, 9H), 0.65 (dd, J = 16.7, 7.9 Hz, 9H).

Compound 4-1 (Qx-1-CHO): Compounds 3-1 (200 mg; 0.163mmol) were dissolved into 1,2-dichloroethane (20 ml) in a three-neck flask. The solution was flushed with nitrogen in 0°C for 30 min. Then, add $POCl_3$ (0.25 mL) and DMF (0.25 mL) to the solution, then let the solution temperature return to room temperature for 1h. Next, the solution was reacted at 85 °C for 16 h under nitrogen protection. After that the mixture was poured into ice water (50 mL), neutralized with aqueous AcONa. Washed with saturated salt water and dichloromethane. The solvent was removed under reduced pressure. The crude product was subsequently purified by column chromatography on silica gel to afford compound 4-1 as orange solid (176mg, 84% yield). ^{1}H NMR (400 MHz, CDCl3) δ 10.18 (s, 2H), 7.85 – 7.80 (m, 4H), 7.48 – 7.43 (m, 6H), 4.74 (t, J = 11.6 Hz, 4H), 3.25 (t, J = 7.6 Hz, 4H), 1.95 (dd, J = 14.9, 7.6 Hz, 4H), 1.45 – 1.21 (m, 36H), 1.14 – 0.86 (m, 36H), 0.70 – 0.64 (m, 12H).

Compound 4-2 (Qx-2-CHO): was synthesized by similar procedure as compound 4-1 with compound 3-2. The final product were obtained as orange solid (184mg, 88% yield). ^{1}H NMR (400 MHz, CDCl3) δ 10.21 (s, 2H), 9.73 (dd, J = 8.0, 1.1 Hz, 2H), 8.74 (d, J = 8.1 Hz, 2H), 7.93 (ddd, J = 15.1, 11.5, 4.5 Hz, 4H), 4.76 (d, J = 7.9 Hz, 4H), 3.35 (t, J = 7.6 Hz, 4H), 2.09 – 1.98 (m, 4H), 1.63 – 1.19 (m, 36H), 1.19 – 0.90 (m, 27H), 0.89 – 0.80 (m, 9H), 0.70 – 0.59 (m, 12H).

Qx-1: Compound 4-1 (176mg, 0.137mmol) and compound 5 (189mg, 0.822mmol) were dissolved into

dry chloroform (60mL) in a three-neck flask. The solution was flushed with nitrogen for 20 min. After 0.8mL pyridine were added, the mixture was stirred at 65°C overnight. After cooling to room temperature, the reaction mixture was poured into water and extracted several times with chloroform. Then the solvent was removed under reduced pressure, and the crude product was purified by column chromatography on silica gel to yield Qx-1 as black solid (183mg, 78% yield). ^{1}H NMR (400 MHz, CDCl3) δ 9.20 (s, 2H), 8.60 (dd, J = 9.8, 6.5 Hz, 2H), 7.80 (d, J = 3.7 Hz, 4H), 7.72 (t, J = 7.5 Hz, 2H), 7.47 (s, 6H), 4.84 (s, 4H), 3.29 (s, 4H), 2.21 (s, 2H), 1.90 (s, 4H), 1.53 – 0.80 (m, 64H), 0.80 – 0.60 (m, 12H). MALDI-TOF MS (m/z): 1710.082.

Qx-2: was synthesized by similar procedure as Qx-1 between compound 4-2 and compound 5. The final product were obtained as black solid (196mg, 80% yield). ^{1}H NMR (400 MHz, CDCl3) δ 9.50 – 9.43 (m, 2H), 9.01 (s, 2H), 8.64 (d, J = 7.5 Hz, 2H), 8.31 – 8.21 (m, 2H), 7.95 – 7.85 (m, 4H), 7.55 (dd, J = 9.6, 4.8 Hz, 2H), 4.95 (d, J = 7.6 Hz, 4H), 3.25 (t, J = 7.0 Hz, 4H), 2.42 (s, 2H), 1.91 (dt, J = 15.1, 7.7 Hz, 4H), 1.55 – 0.89 (m, 64H), 0.87 – 0.61 (m, 18H). MALDI-TOF MS (m/z): 1707.694.

We have provided the solubility of Qx-1, Qx-2 and Y6 through test the UV-vis absorption spectra as shown in Supplementary Figure 3 and Supplementary Table 1. Firstly, we obtained the linear relationship between concentration and UV-vis absorption intensity by measuring the UV-vis absorption spectra of chloroform solutions with different known concentrations, and then further measured the UV-vis absorption spectra of the diluted saturated solution, and deduced the solubility of the material in chloroform. From the experimental data, we obtained the solubility of Qx-1, Qx-2, and Y6 as 36.2mg/ml, 13.9 mg/ml, and 28.2mg/ml, which is beneficial for device fabrication.

As shown in Supplementary Figure 4b, after thermal annealing the films of Qx-1 and Qx-2 produce an obvious red shift, which indicates that thermal annealing can enhance molecular stacking and

stronger π-π intermolecular interaction. In addition, the extinction coefficients of the acceptors and the blend film absorption with PM6 are shown in Supplementary Figure 4c-d. Although Qx-1 exhibits a higher absorbance coefficient near 800nm, Qx-2 has a stronger absorbency near 650 nm, so these two acceptors have obtained comparable short-circuit current (J_{sc}) parameters in devices when blend with PM6.

As shown in Supplementary Figure 5, Both Qx-1 and Qx-2 mainly show the tightly overlapping packing mode of end groups-intermediate cores (EG-core; dimer 1) and the parallel packing mode of end groups-end groups (EG-EG; dimer 2), which build good transmission channel for the transfer of charge transport. Compared with other acceptors‘ crystal structures have reported, such as Y6, L8-BO, and other Y-system acceptors all lead to 3D interpenetrating networks,[17, 18] whereas Qx-1 and Qx-2 all show traditional 1D connections in the crystal. Traditional concepts believe that 3D interpenetrating networks mode is more conducive to the transfer of charges than 1D connections mode. However, in this work, come up with a counter-intuitive conclusion, it is proved that any stacking mode that can transport perpendicularly to the interface layer is beneficial in the active layer of OSC. The backbone of Qx-1 exhibits a little non-planar structure, due to the steric hindrance between the two benzene rings in the core.

We have provided independent certification of PM6:Qx-1and PM6:Qx-2 from the National Institute of Metrology (NIM), and the results are shown in Supplementary Figure 11. With an area of 2.558 mm^2 mask, the device based on PM6:Qx-1 shows PCE of 17.6 % with V_{oc} of 0.880 V, J_{sc} of 0.658 mA (25.72 mA cm^{-2}), and FF of 77.6%; the device based on PM6:Qx-2 shows PCE of 17.5 % with V_{oc} of 0.887 V, J_{sc} of 0.654 mA (25.57 mA cm^{-2}), and FF of 77.1%. The results are generally consistent with our results. The slight decrease in PCE is probably due to the ambient influence on the devices since

our devices were transferred without any encapsulation. To further verify the photovoltaic performance of our devices, we have also tested the device of PM6:Qx-2 both in our lab and Prof. Zhishan Bo's lab (Beijing Normal University, Beijing 100875, P. R. China, zsbo@bnu.edu.cn).

Supplementary Figure 15a shows that these small molecule acceptor devices all had α values over 0.990, and the bimolecular recombination in the device can be ignored. Besides, in Supplementary Figure 15b, the V_{oc} and $\ln(P_{light})$ can be fitted to a straight line. The slope of the fitted line of these small molecule receptor devices were all close to kT/q ($1.136kT/q$); hence, the trap-assisted recombination can be suppressed. The exciton dissociation and charge collection of devices were investigated through the dependence of the photocurrent density (J_{ph}) on the effective voltage (V_{eff}) (Supplementary Figure 15c).[14] Under short-circuit conditions, the ratio values of exciton dissociation based on Qx-1 and Qx-2 were 97.6%, 97.0%, respectively. These two systems are conducive to obtaining efficient exciton dissociation and charge extraction.

The E_{loss} in organic solar cells can be divided into three parts [19] as follows:

$$E_{\mathrm{loss}} = E_{\mathrm{g}}^{\mathrm{pv}} - qV_{\mathrm{OC}} \tag{9}$$

$$= (E_{\mathrm{g}}^{\mathrm{pv}} - qV_{\mathrm{OC}}^{\mathrm{SQ}}) + (qV_{\mathrm{OC}}^{\mathrm{SQ}} - qV_{\mathrm{OC}}^{\mathrm{rad}}) + (qV_{\mathrm{OC}}^{\mathrm{rad}} - qV_{\mathrm{OC}}) \tag{10}$$

$$= (E_{\mathrm{g}}^{\mathrm{pv}} - qV_{\mathrm{OC}}^{\mathrm{SQ}}) + q\Delta V_{\mathrm{OC}}^{\mathrm{rad,\ below\ gap}} + q\Delta V_{\mathrm{OC}}^{\mathrm{non\text{-}rad}} \tag{11}$$

$$= q\Delta V_1 + q\Delta V_2 + q\Delta V_3 \tag{12}$$

$$= \Delta E_1 + \Delta E_2 + \Delta E_3 \tag{13}$$

$$E_{\mathrm{g}}^{\mathrm{pv}} = \frac{\int_{\mathrm{a}}^{\mathrm{b}} E_{\mathrm{g}} . P(E_{\mathrm{g}}) . \mathrm{d}E_{\mathrm{g}}}{\int_{\mathrm{a}}^{\mathrm{b}} P(E_{\mathrm{g}}) . \mathrm{d}E_{\mathrm{g}}} \tag{14}$$

In this formula, q is the elementary charge; $V_{\mathrm{oc}}^{\mathrm{SQ}}$ is the maximum voltage in the Shockley–Queisser (SQ) limit model, and $V_{\mathrm{oc}}^{\mathrm{rad}}$ is the open-circuit voltage with only radiative recombination in the device. The integral boundaries a and b are selected where P(a) = P(b) = 0.5max[P(E_g)]. The selection of

integral boundaries serves to exclude the influence of noisy data and negative value of P(Eg), and is not physically motivated. While the factor 0.5 in the in the choice of a and b is fairly arbitrary, slightly different choices would not strongly affect the result except for very noisy data[20].

Supplementary Figure 18b-f shows increasing the temperature of thermal annealing also did not stimulate the crystallinity of Qx-1 in the blend film. As shown in Supplementary Figure 20d-e and Supplementary Figure 20g-h, the Qx-1 and Qx-2 blend films exhibit clearly fibrous structures in AFM images with root-mean-square (RMS) values of 1.66 and 0.898 nm, which is beneficial for charge transportation and achieve higher J_{sc} and FF.[21, 22] It can be seen in the neat films in Supplementary Figure 20a-c that the fibrous structures in the blend films is mainly derived from the aggregation characteristics of PM6. The non-planar structure and poor miscibility of Qx-1 lead to greater roughness; on the contrary, Qx-2 planer structure and better miscibility result in less roughness. The TEM of the optimal blend films are shown in Supplementary Figure 20f and Supplementary Figure 20i. Consistent with the results of AFM, the planar structure of Qx-2 is more conducive to breaking the strong self-aggregation of PM6.

Resonance soft X-ray scattering (R-SoXS) was used to detect the phase separation information of the two systems (Supplementary Figure 21). We obtained the high-contrast scattering information of these organic materials under 285.2 eV X-rays. The PM6:Qx-1 and PM6:Qx-2 systems showed scattering peaks at q = 0.07 and 0.15 nm^{-1}, respectively, indicating that the phase region size of PM6: Qx-2 (21.63 nm) is smaller than that of PM6:Qx-1 (43.56 nm). However, the phase purity of PM6:Qx-2 system is lower than that of PM6:Qx-1, which is 0.91 and 1, respectively.

Transient absorption spectroscopy (TAS) is used to further explore the diffusion behavior of exciton and charge dynamics by exciton-exciton annihilation (EEA) method.[23, 24] The diffusion behavior of

exciton and charge dynamics by exciton-exciton annihilation (EEA) method.[23, 24] The radiative and non-radiative deactivations with an intrinsic exciton lifetime constant (k) and the bimolecular exciton-exciton annihilation with a bimolecular decay rate coefficient (γ) were considered to be the two main quenching channels of extion. The values of k and γ can be used to calculate the exciton diffusion coefficient (D).

We further investigate the photo-induced charge transfer (CT) process of the active layer by measuring the transient absorption (TA) spectra of the neat and blend film, as shown in Supplementary Figure 24 and Supplementary Figure 25. Pump wavelength was set to 400nm and 800nm to selectively excite the donor and acceptors in blend films. The TA images with a pump wavelength of 800nm in the VIS and NIR regions of these blend films are shown in Supplementary Figure 25. As the traces of donor and acceptors can be well separated, the hole transfer dynamics can be extracted near 630nm. The blend films of Qx-2 exhibited a faster hole transfer rate, the blend film of Qx-1 is the second fastest, the blend film of Y6 is the slowest. This result agrees with the higher mobility of Qx-2.

Supplementary Figures

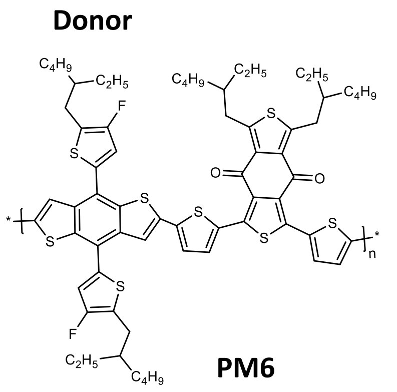

Supplementary Figure. 1 The chemical structures of PM6 polymer donor.

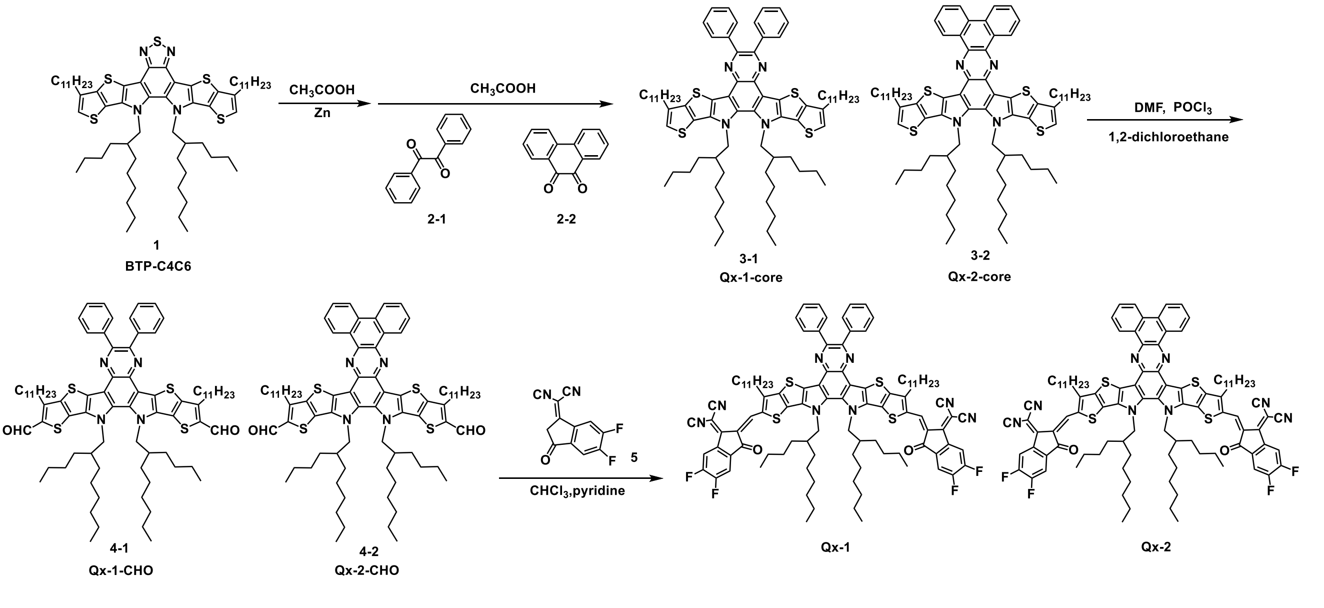

Supplementary Figure. 2 Synthetic routes of Qx-1 and Qx-2.

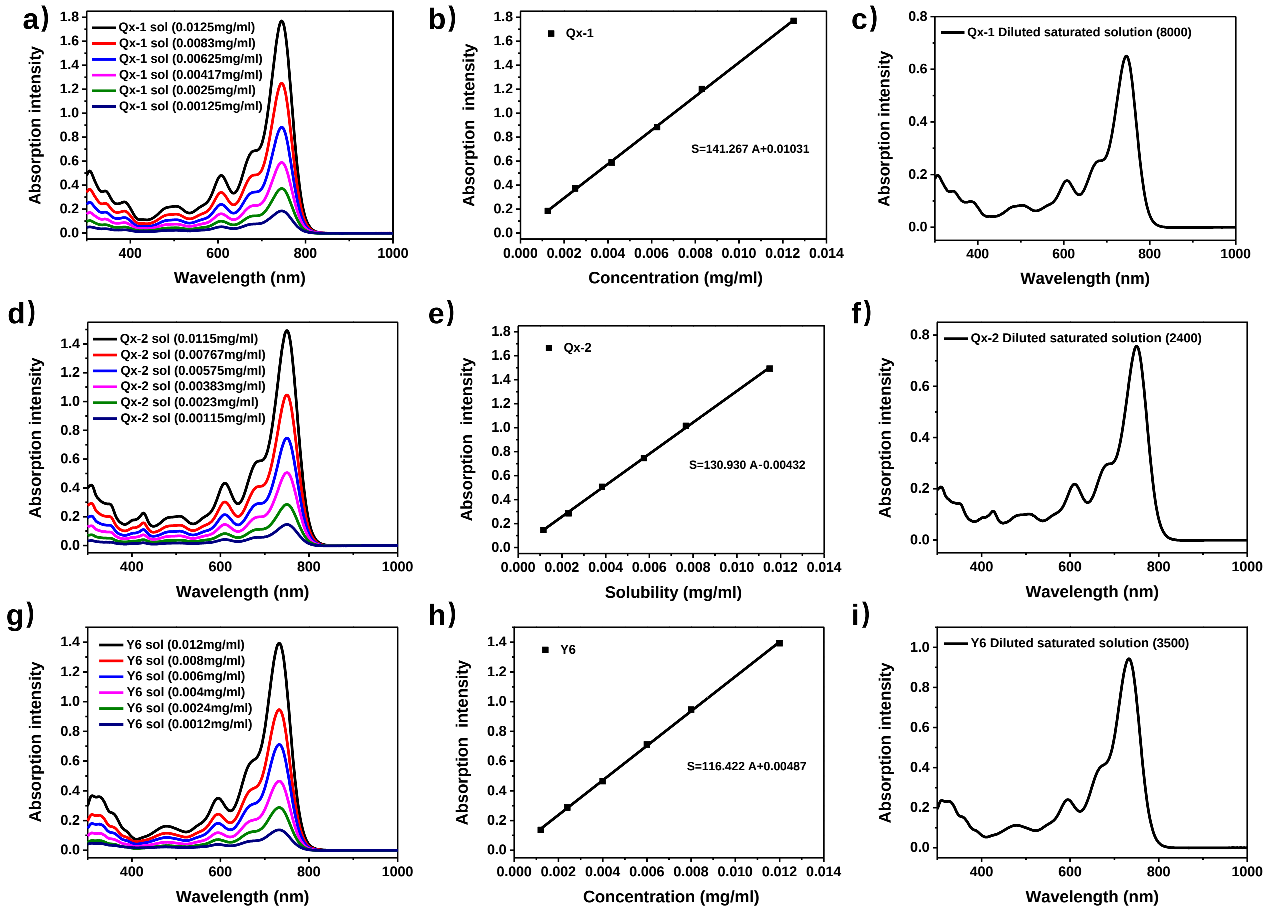

Supplementary Figure. 3 The solubility of Qx-1, Qx-2, and Y6 in chloroform**.** (a, d, g) UV-vis absorption spectra of acceptors of chloroform solutions with different known concentrations; (b, e, h) the linear relationship between concentration and UV-vis absorption intensity; (c, f, i) the UV-vis absorption spectra of the diluted saturated solution.

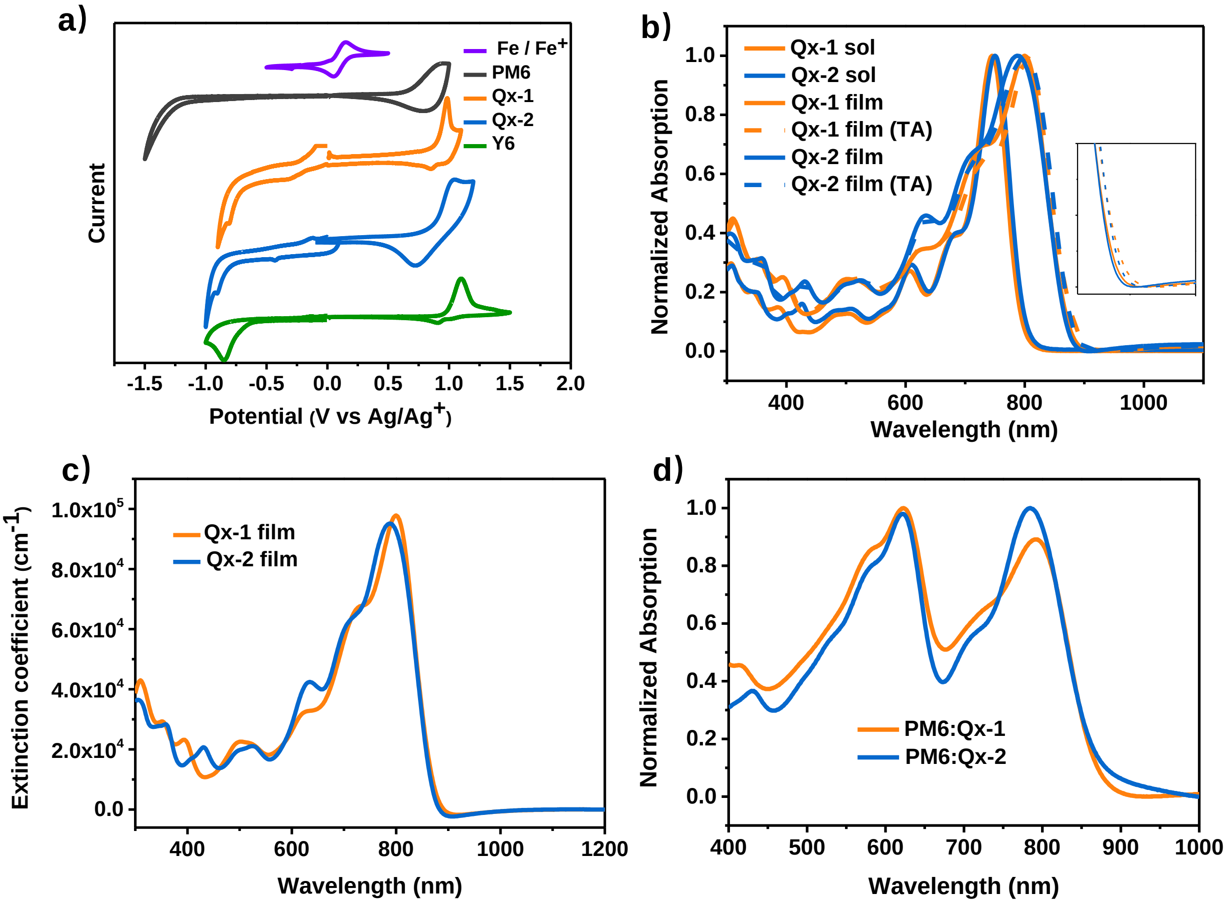

Supplementary Figure. 4 The optoelectronic characterization of acceptors. (a) Electrochemical cyclic voltammetry curves of these acceptors films measured in 0.1 mol $L^{-1}Bu_4NPF_6$ acetonitrile solutions. (b)Normalized absorption spectra of Qx-1, Qx-2 solution, films, and thermal annealing films. (c) Extinction Coefficient in film of Qx-1and Qx-2; (d) Normalized absorption spectra of blend films of Qx-1and Qx-2.

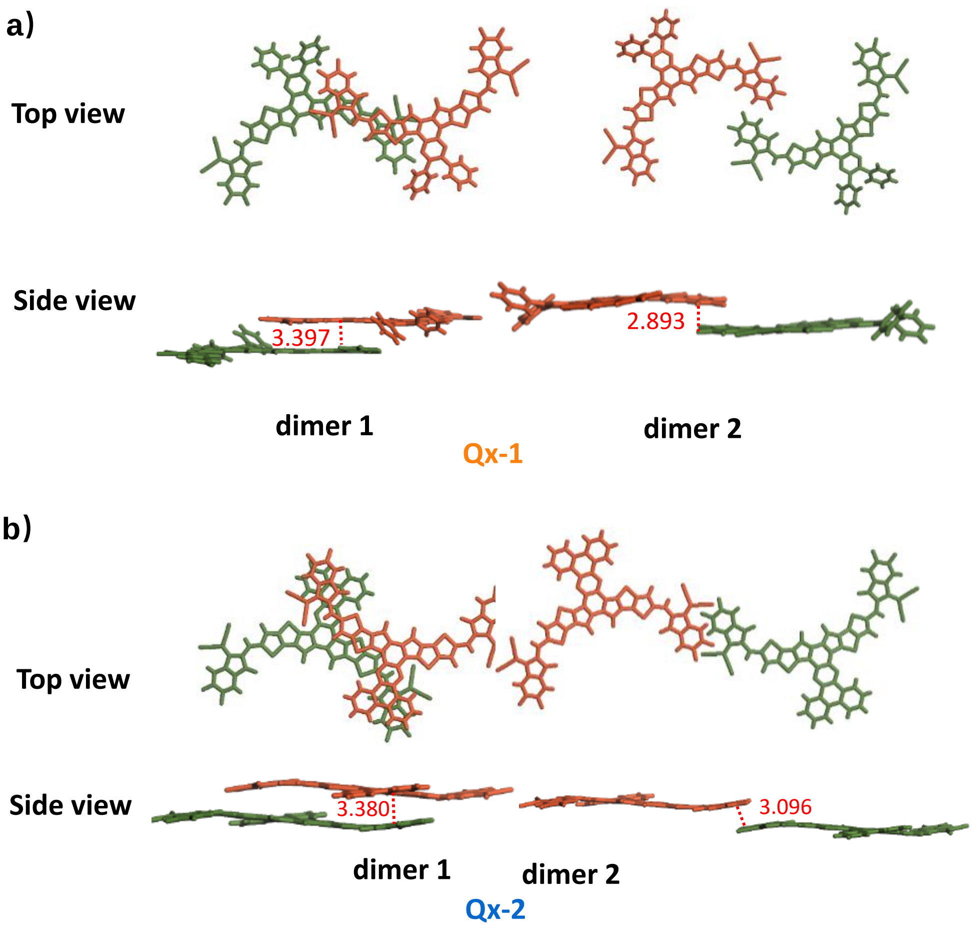

Supplementary Figure. 5 The main view of molecular packing sketch of (a)Qx-1 and (b) Qx-2 according to single-crystal data.

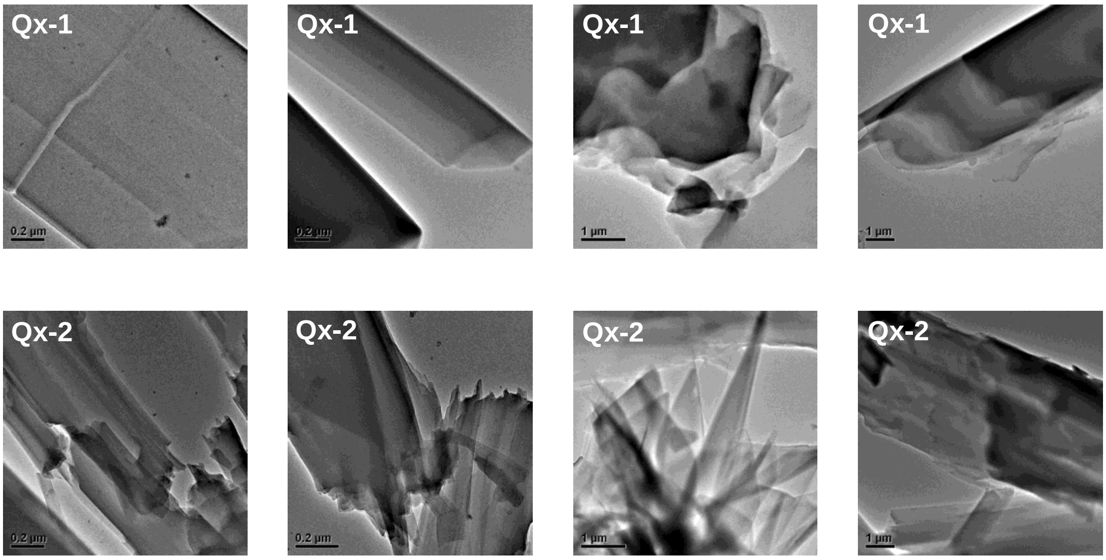

Supplementary Figure. 6 The TEM images of single crystals.

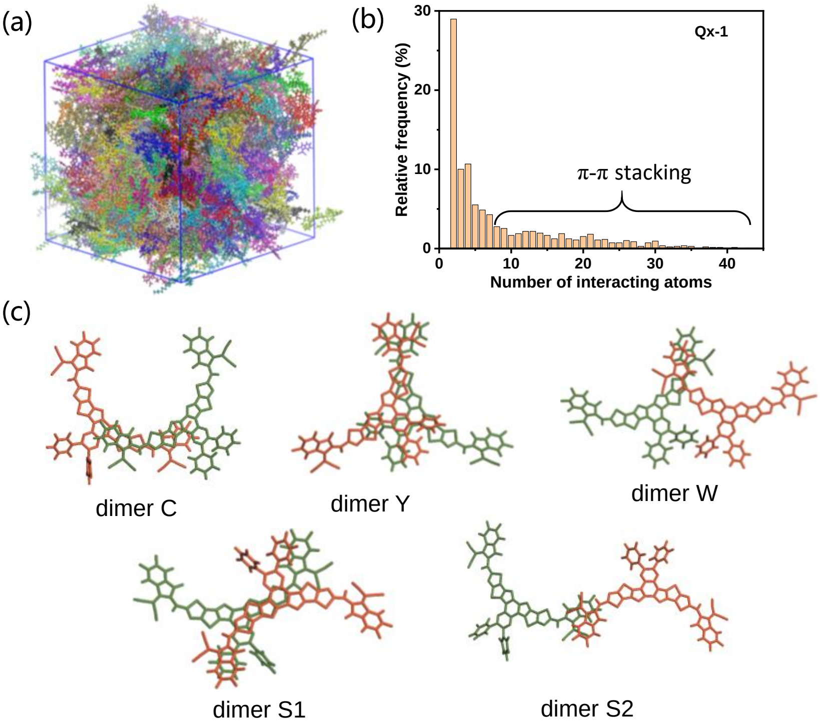

Supplementary Figure. 7 The results of atomistic molecular dynamic (MD) simulations of Qx-1. (a) Representative morphology of the simulated Qx-1 thin film. (b) Statistics of the possible dimers in thin film by MD simulations. The molecular pair with ≥ 8 interacting atoms (inter-atomic distance is smaller than the sum of the of the atomic van der Waals radii) is regarded as π-π stacking. (c) Representative π-π stacking dimers in the Qx-1 film.

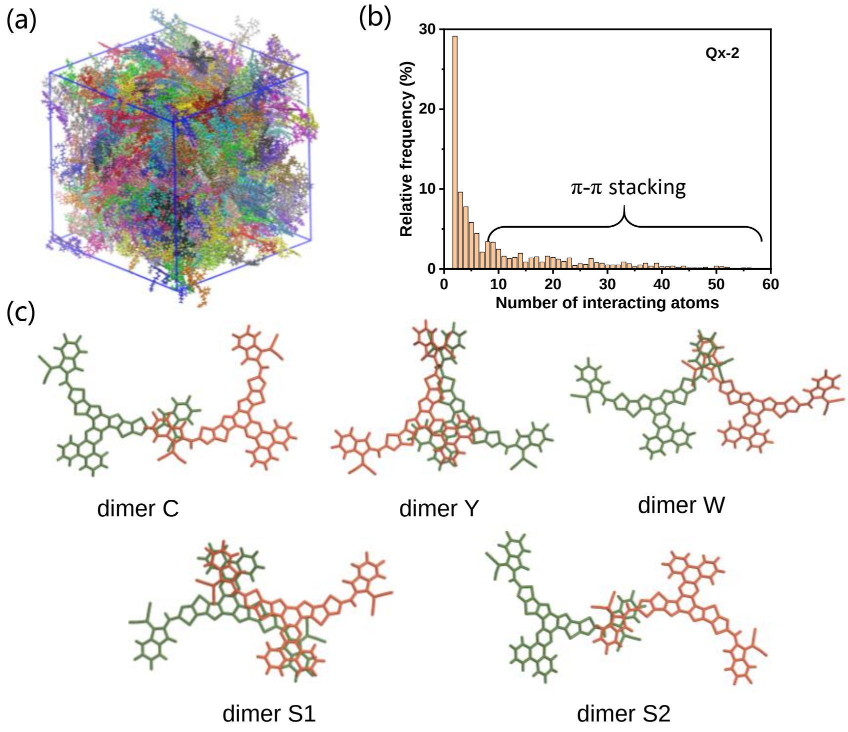

Supplementary Figure. 8 The results of atomistic molecular dynamic (MD) simulations of Qx-2. (a) Representative morphology of the simulated Qx-2 thin film. (b) Statistics of the possible dimers in thin film by MD simulations. The molecular pair with ≥ 8 interacting atoms (inter-atomic distance is smaller than the sum of the of the atomic van der Waals radii) is regarded as π-π stacking. (c) Representative π-π stacking dimers in the Qx-2 film.

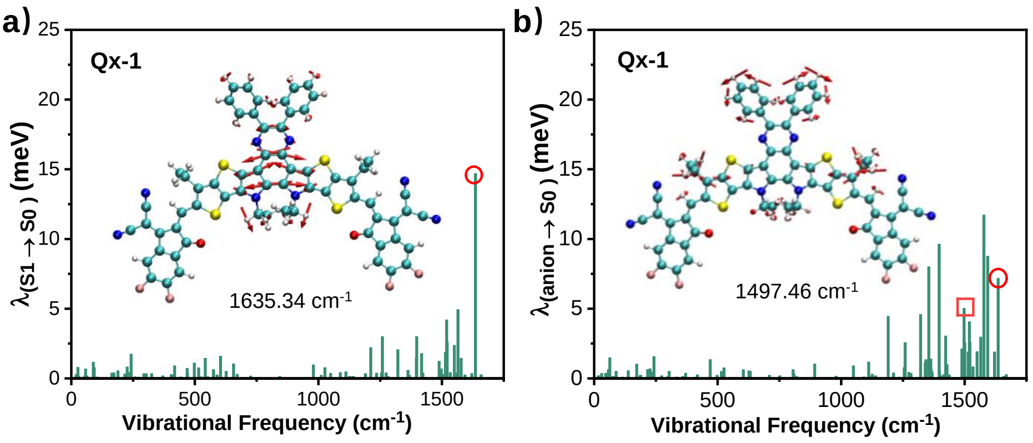

Supplementary Figure. 9 Contributions of each vibrational modes to the reorganization energy for the $S_1 \rightarrow S_0$ and anion $\rightarrow S_0$ transitons in Qx-1 accetpor. Illustration of the displacement vectors for the vibrational normal modes marked by (a) circles (at 1635 cm^{-1}) and (b) squares (at 1497 cm^{-1}) are inserted. The length of displacement vectors stands for the magnitude of vibrational strengh.

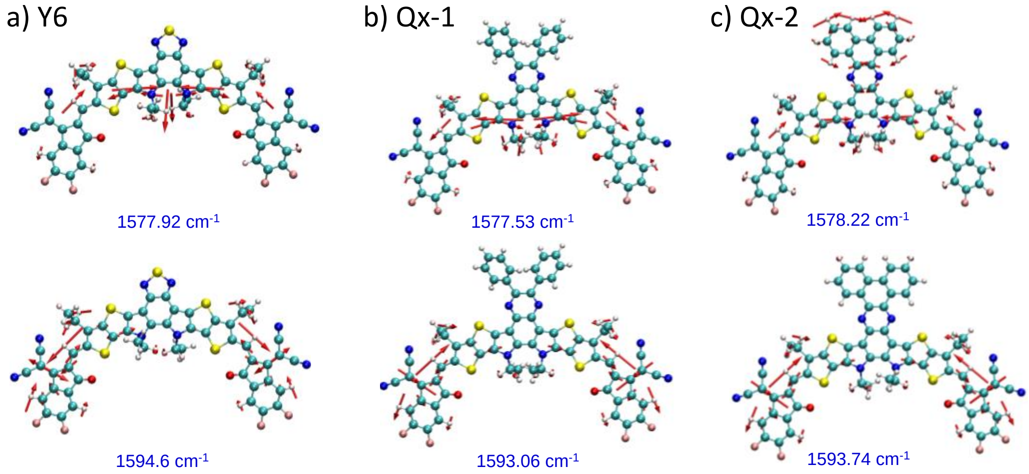

Supplementary Figure. 10 Two other important displacement vectors that contribute to the reorganization energy for anion $\rightarrow S_0$ transitons in (a) Y6, (b) Qx-1 and (c) Qx-2 accetpor. The length of displacement vectors stands for the magnitude of vibrational strengh.

a)

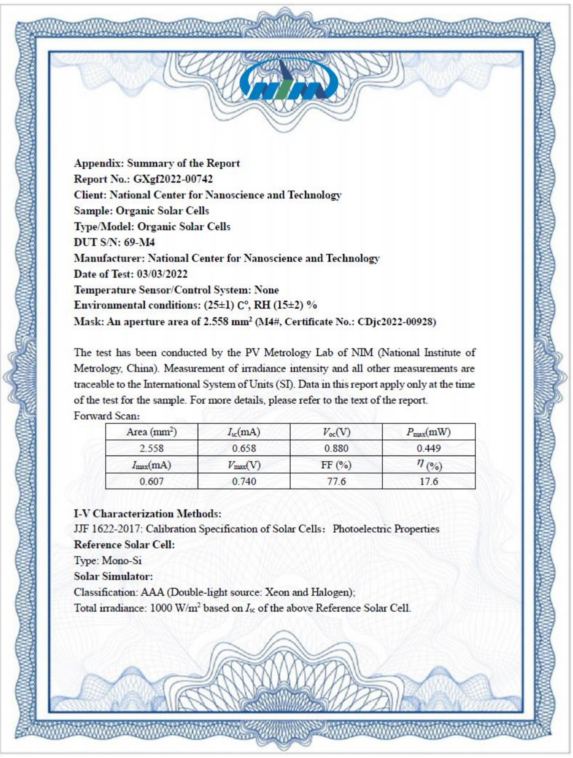

Appendix: Summary of the Report
Report No.: GXgf2022-00742
Client: National Center for Nanoscience and Technology
Sample: Organic Solar Cells
Type/Model: Organic Solar Cells
DUT S/N: 69-M4
Manufacturer: National Center for Nanoscience and Technology
Date of Test: 03/03/2022
Temperature Sensor/Control System: None
Environmental conditions: (25±1) C°, RH (15±2) %
Mask: An aperture area of 2.558 mm^2 (M4#, Certificate No.: CDjc2022-00928)

The test has been conducted by the PV Metrology Lab of NIM (National Institute of Metrology, China). Measurement of irradiance intensity and all other measurements are traceable to the International System of Units (SI). Data in this report apply only at the time of the test for the sample. For more details, please refer to the text of the report.
Forward Scan:

Area (mm^2)	I_{sc}(mA)	V_{oc}(V)	P_{max}(mW)
2.558	0.658	0.880	0.449
I_{max}(mA)	V_{max}(V)	FF (%)	η (%)
0.607	0.740	77.6	17.6

I-V Characterization Methods:
JJF 1622-2017: Calibration Specification of Solar Cells: Photoelectric Properties
Reference Solar Cell:
Type: Mono-Si
Solar Simulator:
Classification: AAA (Double-light source: Xeon and Halogen);
Total irradiance: 1000 W/m^2 based on I_{sc} of the above Reference Solar Cell.

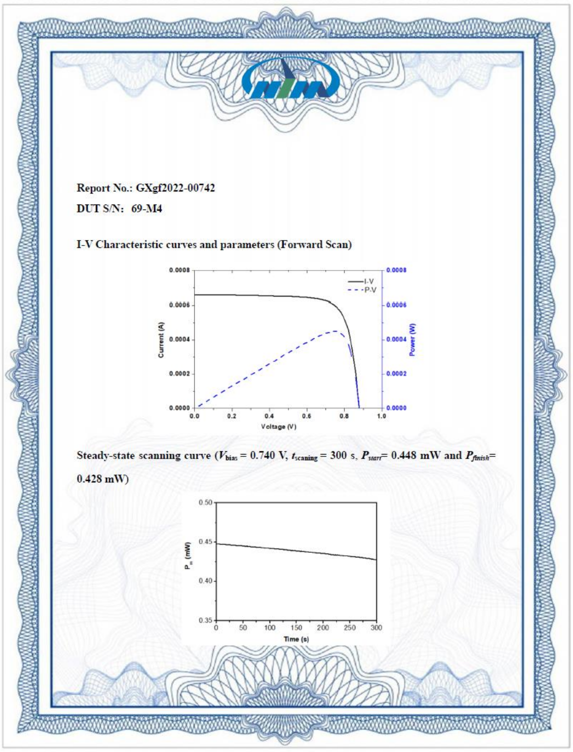

Report No.: GXgf2022-00742
DUT S/N: 69-M4

I-V Characteristic curves and parameters (Forward Scan)

Steady-state scanning curve (V_{bias} = 0.740 V, $t_{scaning}$ = 300 s, P_{start}= 0.448 mW and P_{finish}= 0.428 mW)

b)

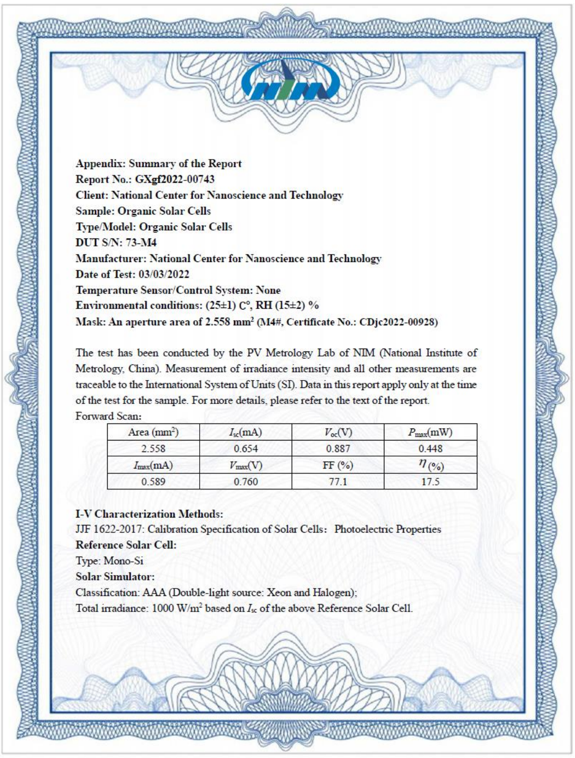

Appendix: Summary of the Report
Report No.: GXgf2022-00743
Client: National Center for Nanoscience and Technology
Sample: Organic Solar Cells
Type/Model: Organic Solar Cells
DUT S/N: 73-M4
Manufacturer: National Center for Nanoscience and Technology
Date of Test: 03/03/2022
Temperature Sensor/Control System: None
Environmental conditions: (25±1) C°, RH (15±2) %
Mask: An aperture area of 2.558 mm^2 (M4#, Certificate No.: CDjc2022-00928)

The test has been conducted by the PV Metrology Lab of NIM (National Institute of Metrology, China). Measurement of irradiance intensity and all other measurements are traceable to the International System of Units (SI). Data in this report apply only at the time of the test for the sample. For more details, please refer to the text of the report.
Forward Scan:

Area (mm^2)	I_{sc}(mA)	V_{oc}(V)	P_{max}(mW)
2.558	0.654	0.887	0.448
I_{max}(mA)	V_{max}(V)	FF (%)	η (%)
0.589	0.760	77.1	17.5

I-V Characterization Methods:
JJF 1622-2017: Calibration Specification of Solar Cells: Photoelectric Properties
Reference Solar Cell:
Type: Mono-Si
Solar Simulator:
Classification: AAA (Double-light source: Xeon and Halogen);
Total irradiance: 1000 W/m^2 based on I_{sc} of the above Reference Solar Cell.

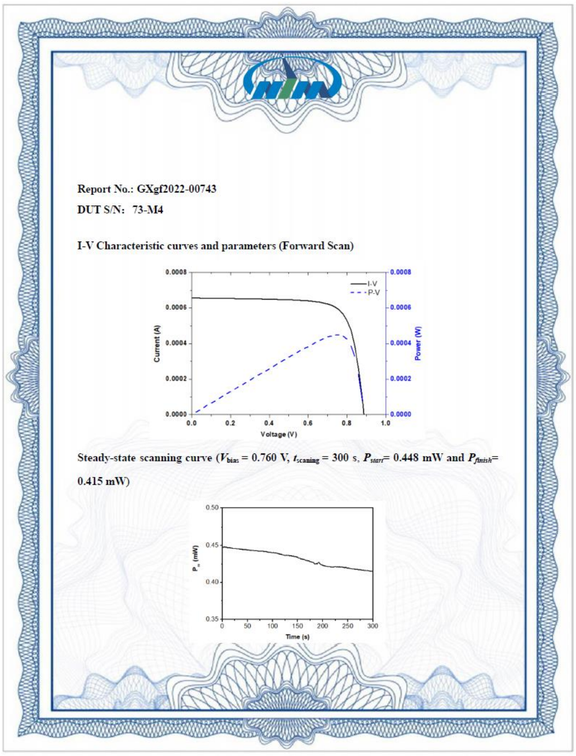

Report No.: GXgf2022-00743
DUT S/N: 73-M4

I-V Characteristic curves and parameters (Forward Scan)

Steady-state scanning curve (V_{bias} = 0.760 V, $t_{scaning}$ = 300 s, P_{start}= 0.448 mW and P_{finish}= 0.415 mW)

Supplementary Figure. 11 Certification report by National Institute of Metrology (NIM), China. (a) PM6:Qx-1 device; (b) PM6:Qx-2 device.

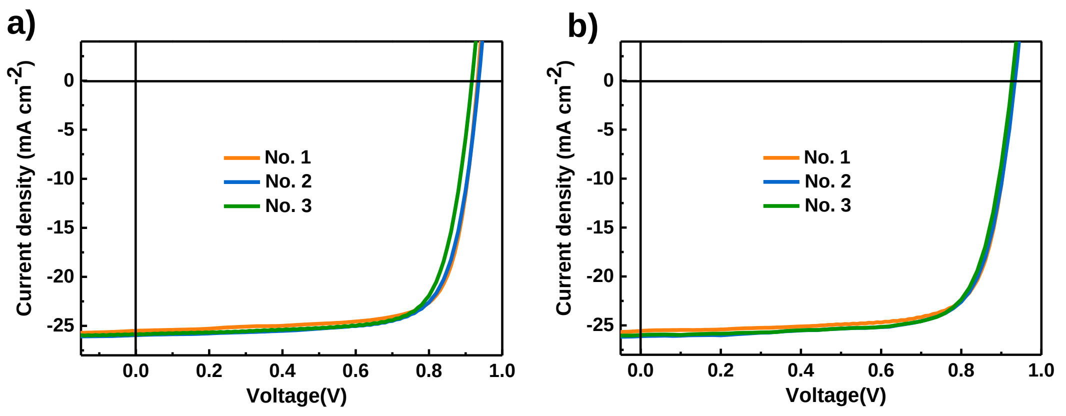

Supplementary Figure. 12 The *J-V* curves of three devices based on PM6:Qx-2. (a) in our lab; (b) in Prof. Bo's lab.

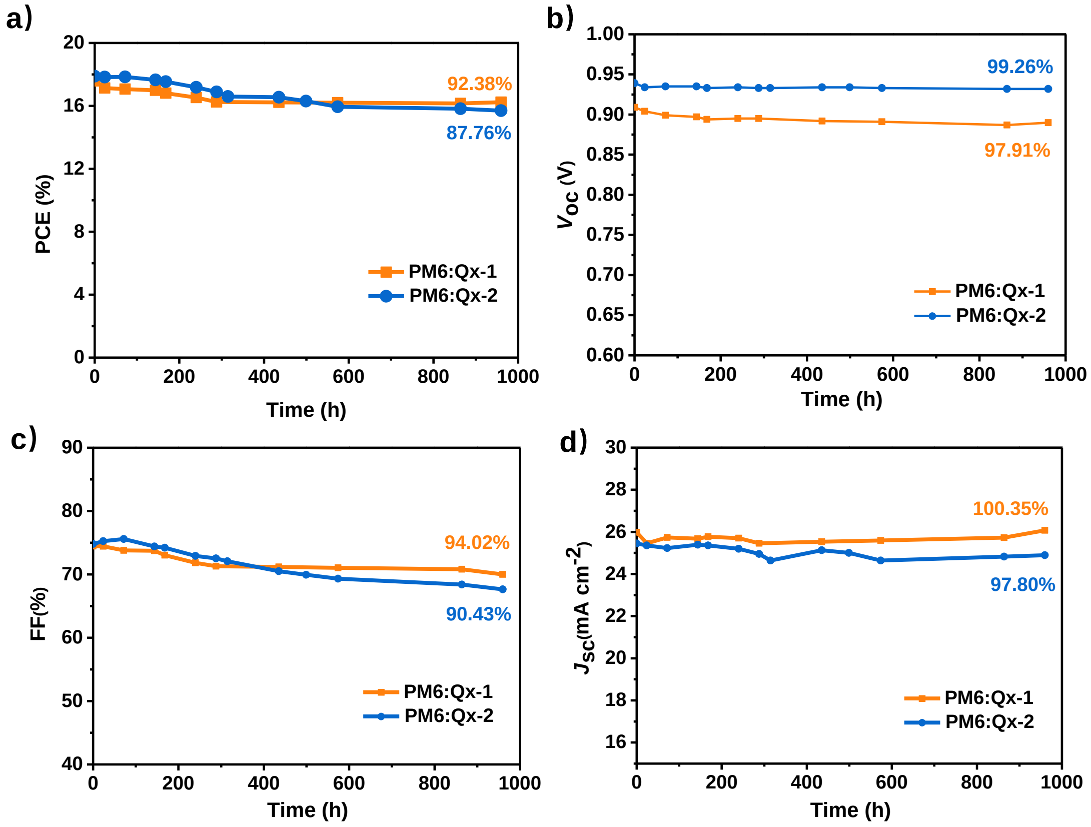

Supplementary Figure. 13 The stability about detailed parameters of placement in glove box with PM6 as the donor. (a) PCE parameters; (b) V_{oc} parameters; (c) FF parameters; (d) J_{sc} parameters.

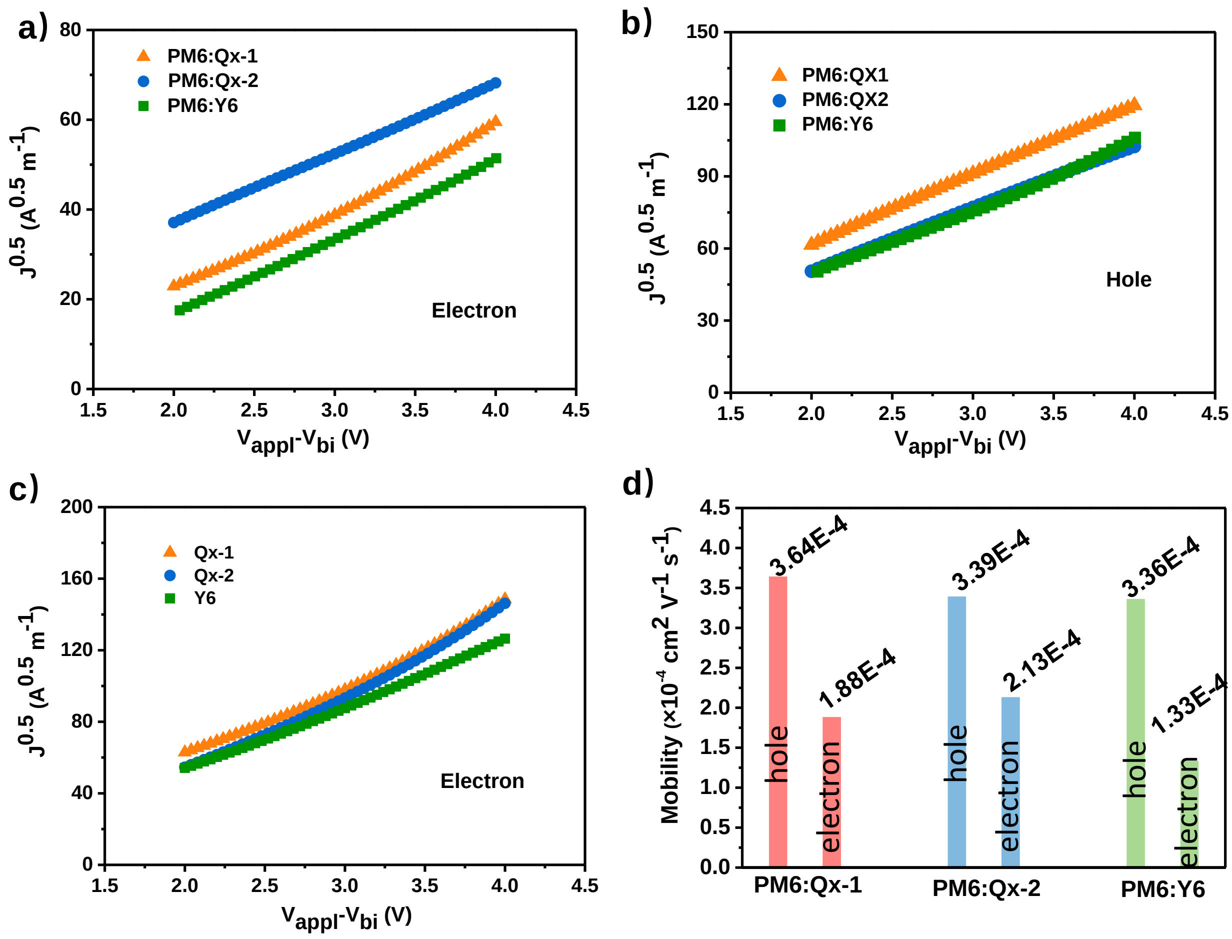

Supplementary Figure. 14 Electron and hole mobility of (a-b;d) the blend films and (c) the neat films.

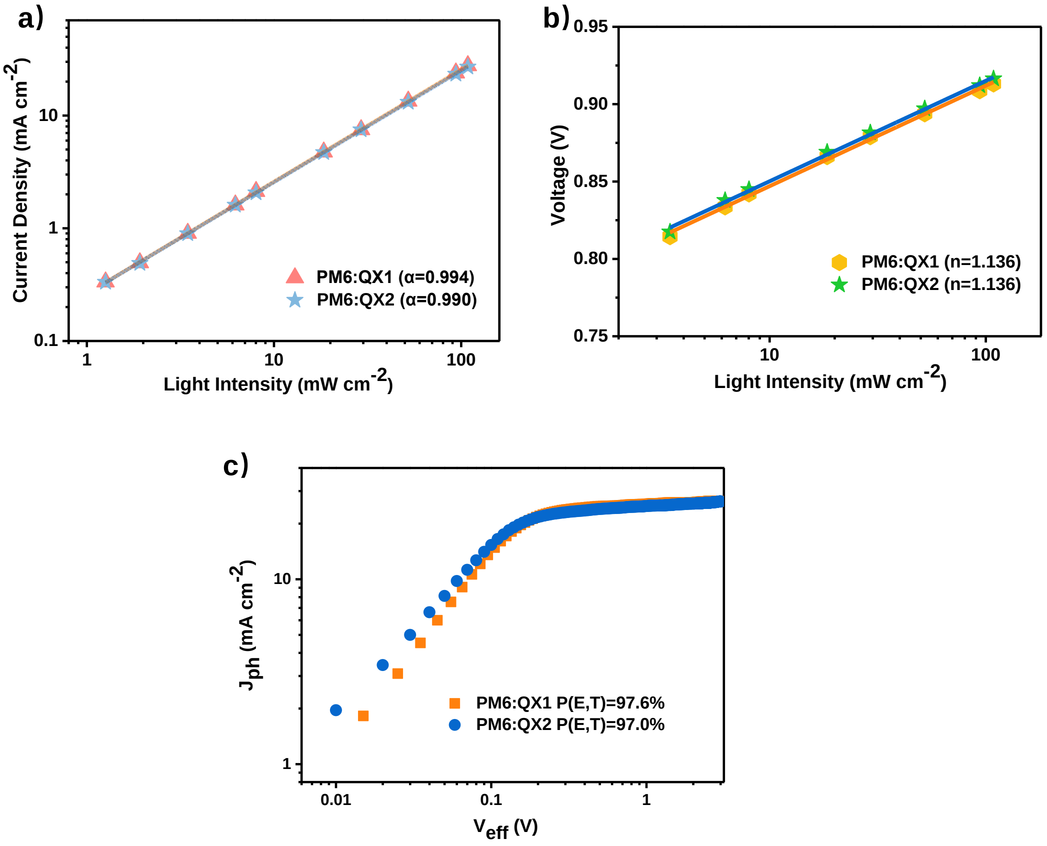

Supplementary Figure. 15 The effect of charge recombination behavior of these devices. (a-b) Light intensity dependence of J_{sc} and V_{oc}; and (c) Dependence of the photocurrent density (J_{ph}) on the effective voltage (V_{eff}).

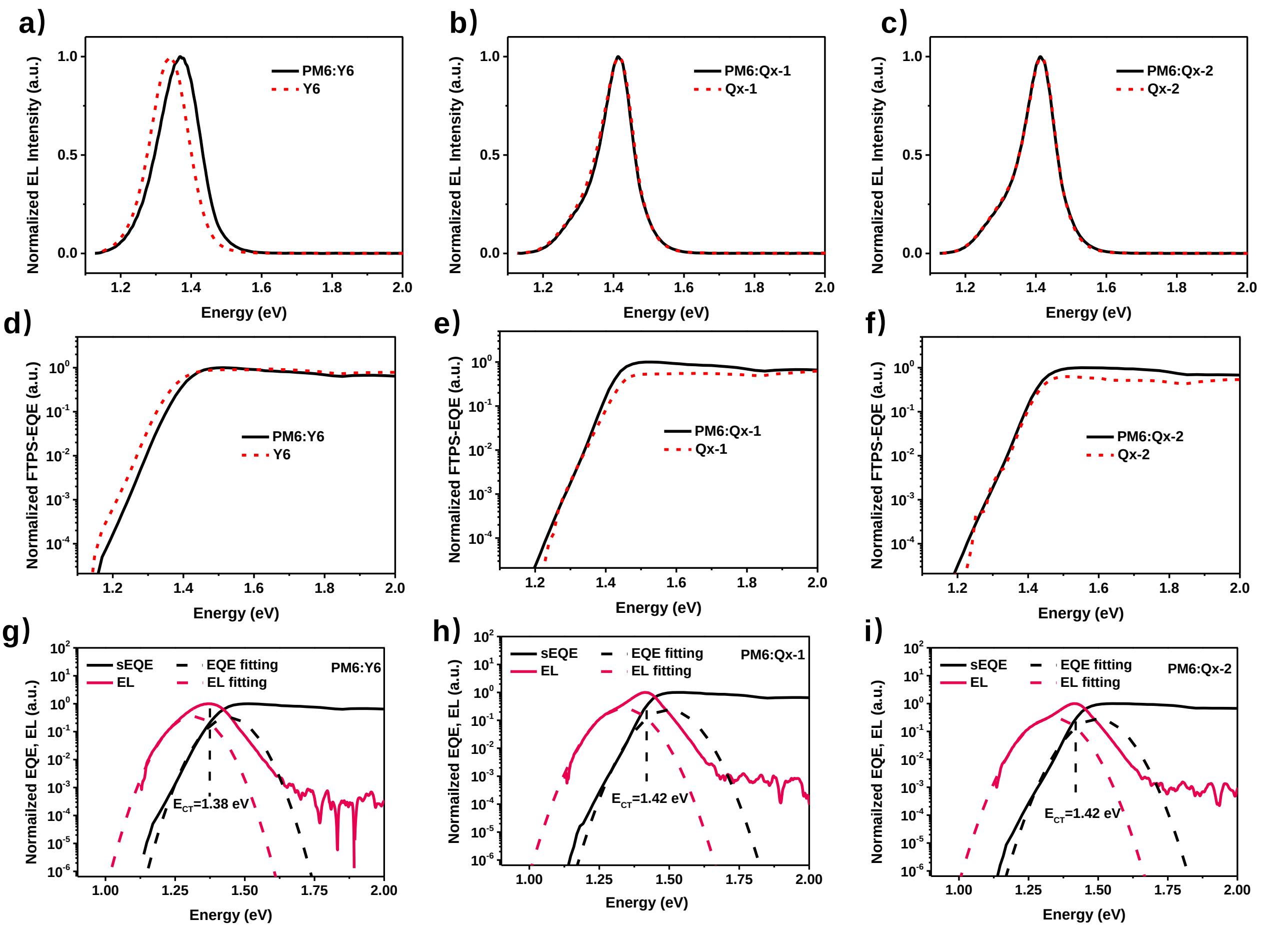

Supplementary Figure. 16 The electroluminescence (EL) spectra and Fourier-transform photocurrent spectroscopy-EQE (FTPS-EQE) spectra. (a-c) Normalized EL curves of blend and corresponding single component solar cells; (d-f) FTPS-EQE spectra of blend and corresponding single component solar cells; (g-i) Normalized FTPS-EQE and EL spectra of the devices.

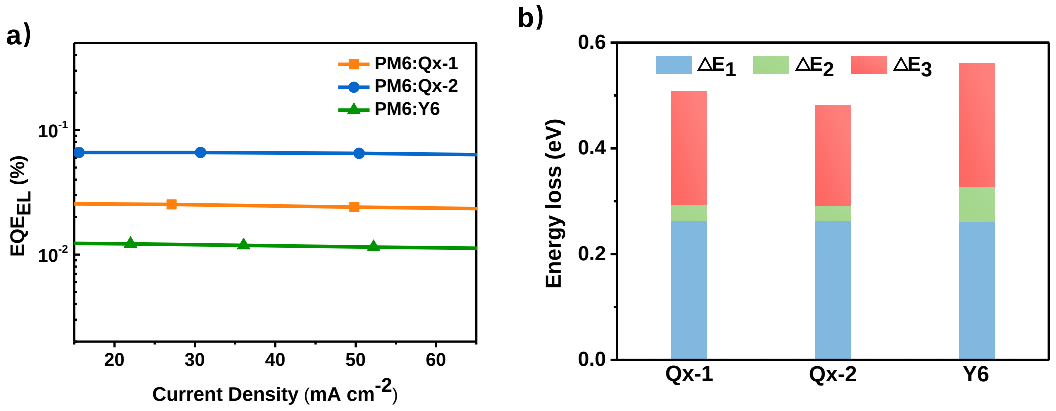

Supplementary Figure. 17 Energy loss of these devices. (a)Electroluminescence quantum efficiency of optimal blend

films at different injected currents; (b) Schematic for the energy loss of solar cells.

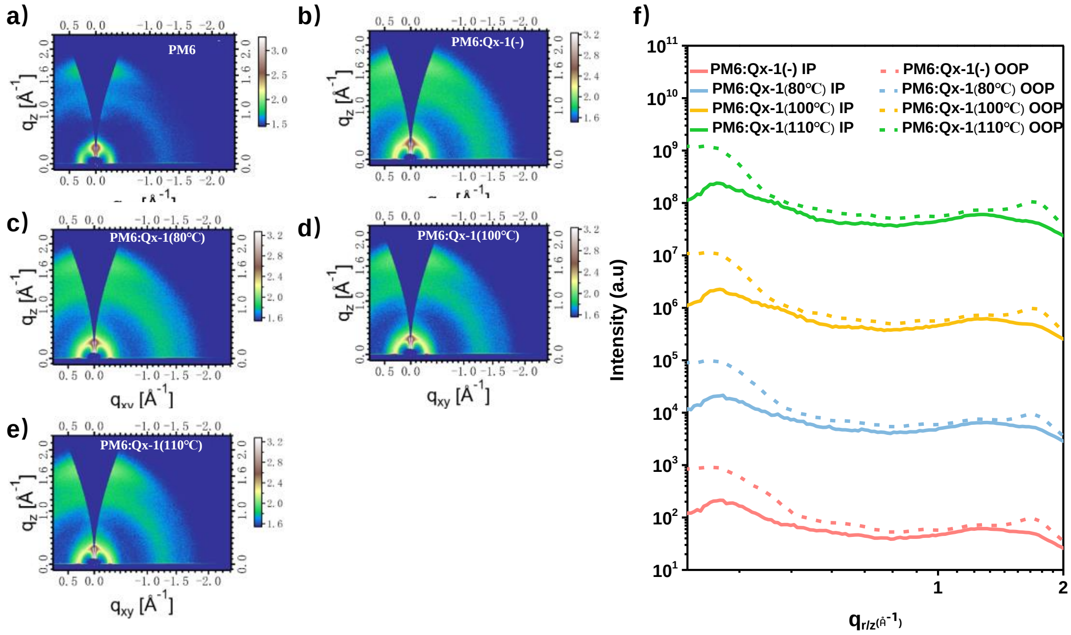

Supplementary Figure. 18 The GIWAXS patterns of PM6 neat film and PM6:Qx-1 blend films with different thermal annealing temperature**.** (a-e) The 2D GIWAXS patterns of the films; (f) In-plane and out-of-plane cuts of the films with GIWAXS patterns.

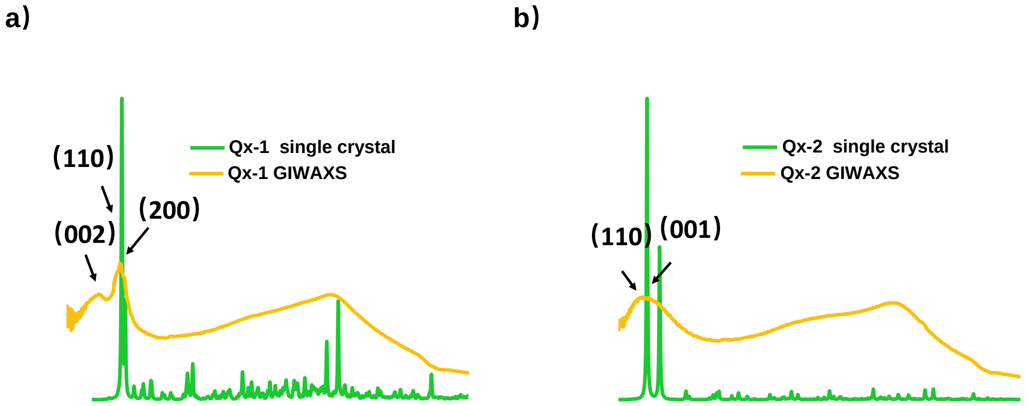

Supplementary Figure. 19 The crystal plane parameter data of single crystal and GIWAXS (a)Qx-1; (b) Qx-2.

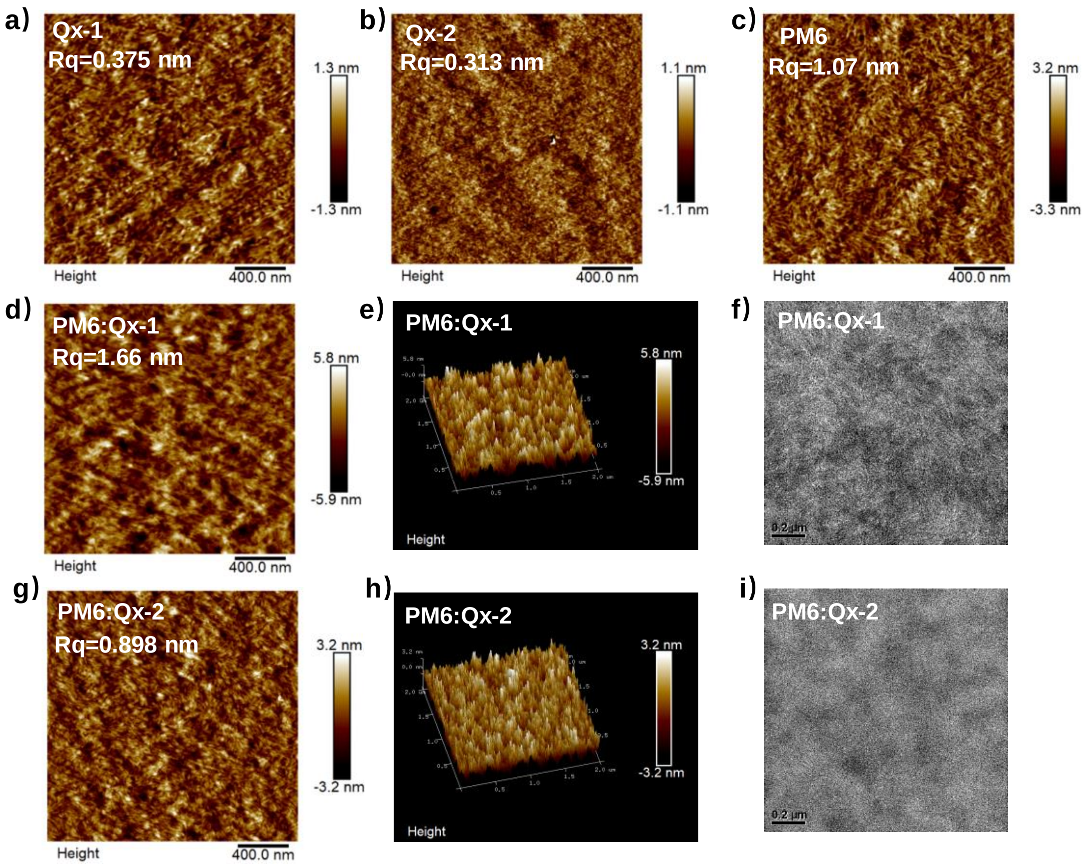

Supplementary Figure. 20 AFM and TEM images. (a-e; g-h) AFM images for neat films and optimal blend films (Rq is the root-mean-square roughness); (f; i) TEM images for optimal blend films.

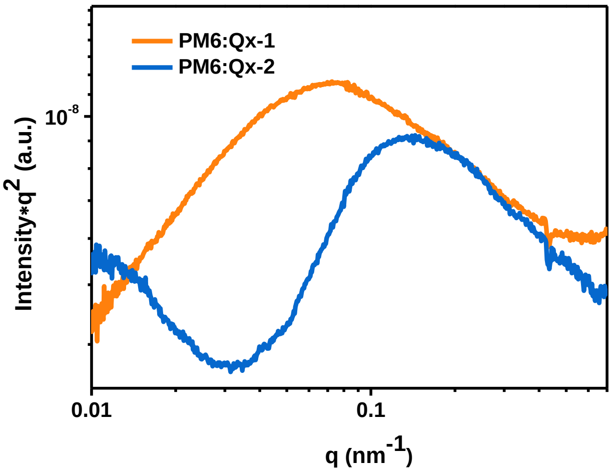

Supplementary Figure. 21 R-SoXS profiles for optimal blend films.

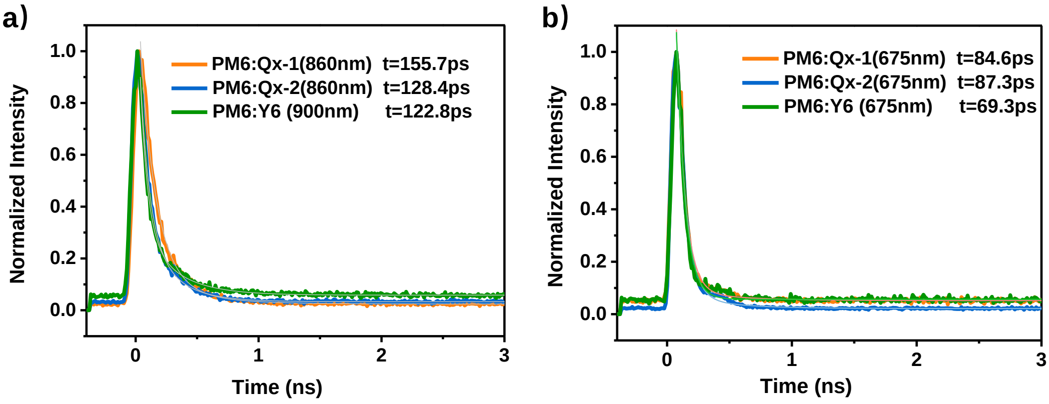

Supplementary Figure. 22 The time-resolution photoluminescence (TRPL) spectrum of blend films. (a) under the probe of 860 nm and 900 nm; (b) under the probe of 675 nm.

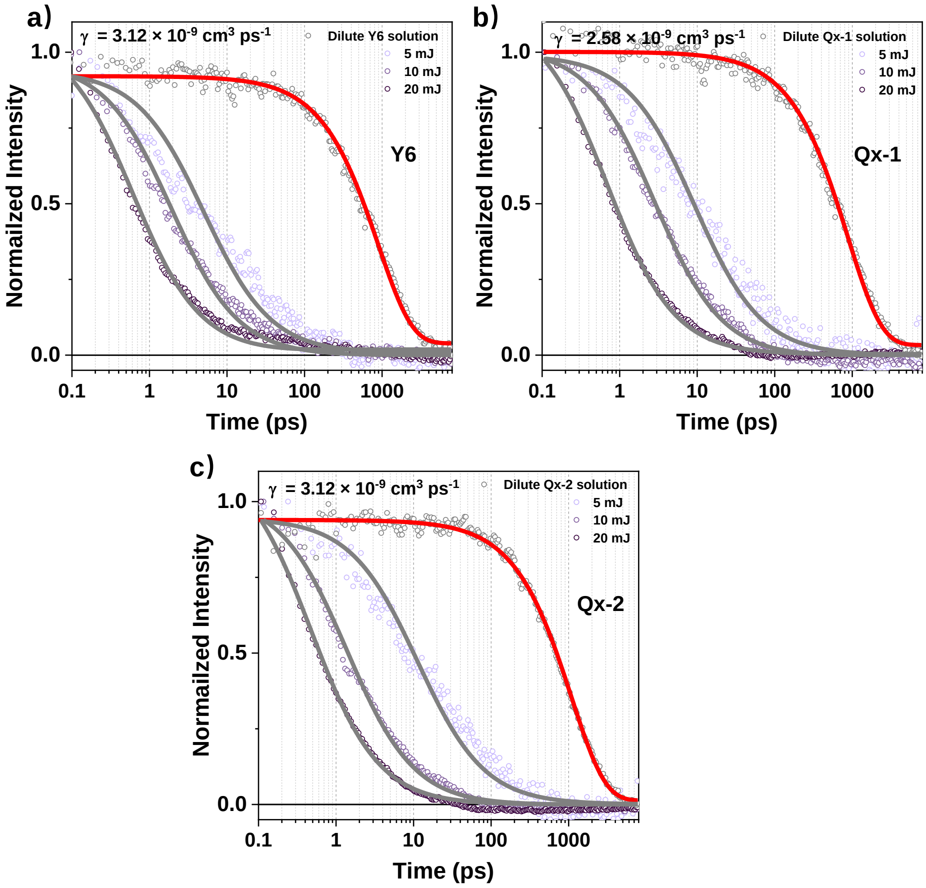

Supplementary Figure. 23 The decay dynamics excitons of 910nm in the dilute solution and the neat films of (a) Y6; (b) Qx-1; and (c) Qx-2 under the excitation of 800 nm with different excitation intensity.

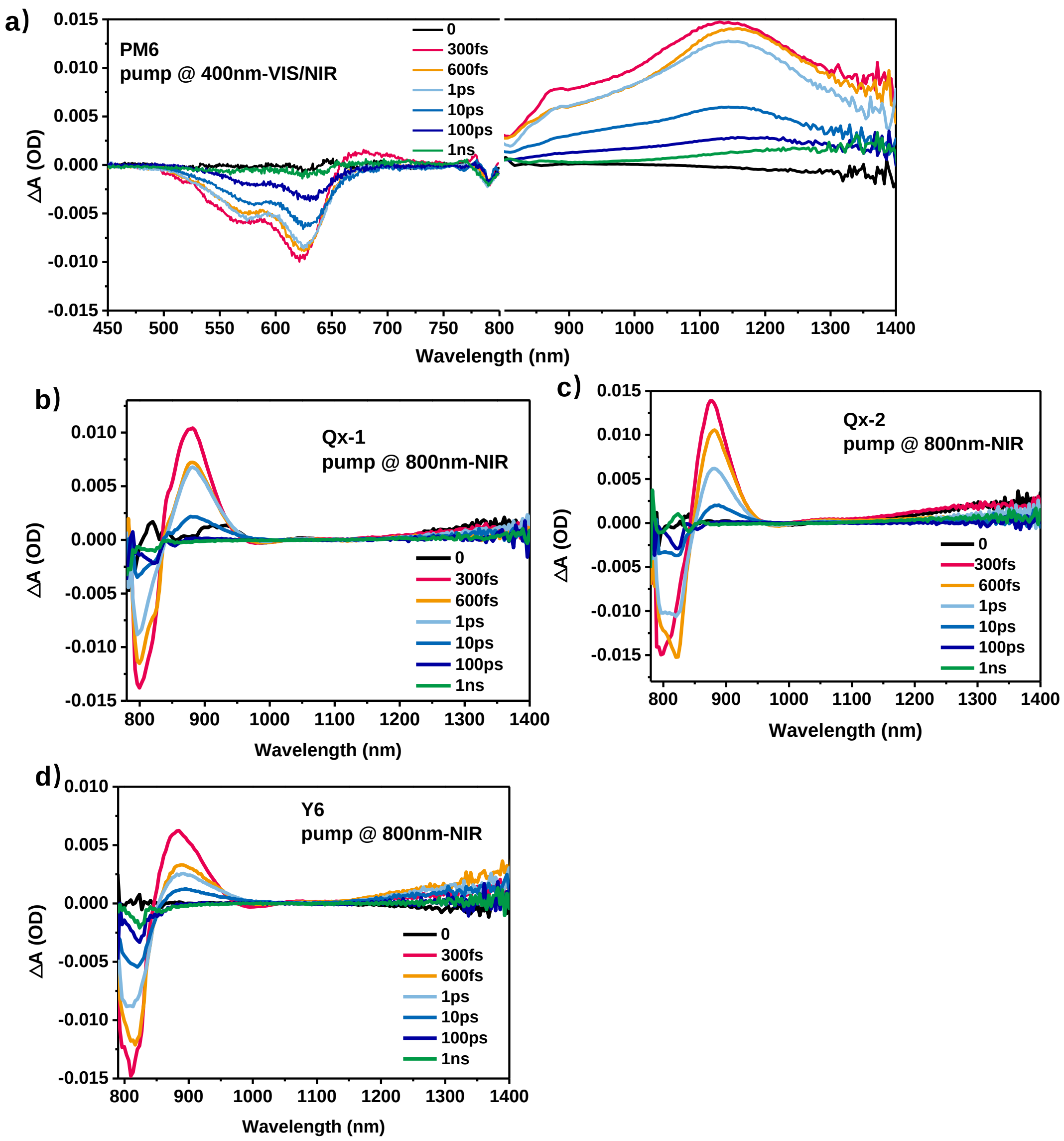

Supplementary Figure. 24 TA spectra of neat films. (a)TA spectra of PM6 film with various decay times under 400 nm excitation; (b-d) TA spectra of neat acceptor films with various decay times under 800 nm excitation.

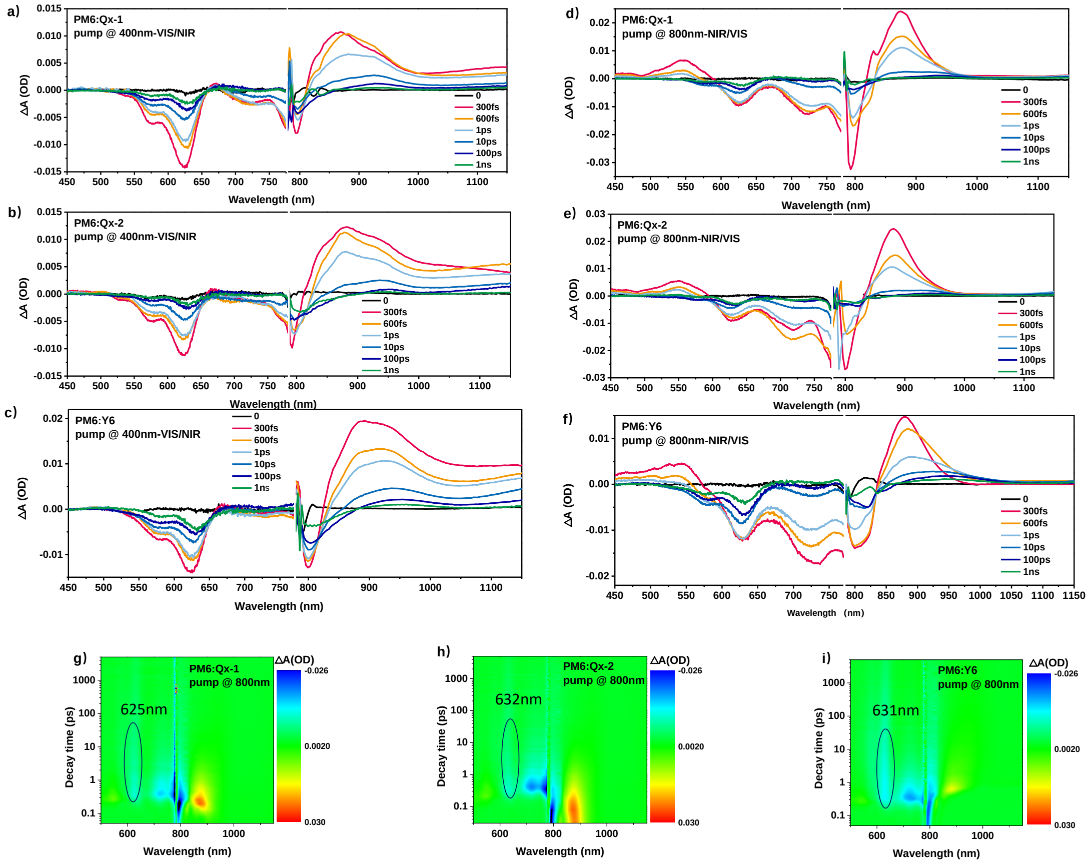

Supplementary Figure. 25 TA spectra of blend films. (a-c) TA spectra of blend films with various decay times under 400 nm excitation; (d-f) TA spectra of blend films with various decay times under 800 nm excitation; (g-i) TA image of blend films with various decay times under 800 nm excitation.

Supplementary Tables

Supplementary Table. 1 UV-vis absorption intensity with different concentrations (in mg/ml).

Qx-1	Standard curve						DSS (*8000)
Concentration	0.0125	0.0083	0.00625	0.00417	0.0025	0.00125	36.2
Absorption intensity	1.77	1.202	0.884	0.589	0.372	0.185	0.65
Qx-2	Standard curve						DSS (*2400)
Concentration	0.0115	0.00767	0.00575	0.00383	0.0023	0.00115	13.9
Absorption intensity	1.492	1.045	0.746	0.506	0.285	0.146	0.756
Y6	Standard curve						DSS (*3500)
Concentration	0.012	0.008	0.006	0.004	0.0024	0.0012	28.2
Absorption intensity	1.393	0.946	0.712	0.465	0.288	0.137	0.943

*DSS= Diluted saturated solution

Supplementary Table. 2 Optical and Electrochemical Properties of Qx-1, Qx-2 and Y6.

Material	λ_{max}^{sol} [a] (nm)	λ_{max}^{film} [a] (nm)	λ_{edge}^{film} [a] (nm)	$E_g^{opt.}$ (TA) (eV)	E_{HOMO} (eV)	E_{LUMO} (eV)
Qx-1	744.86	810.41	911.38	1.36	-5.58	-3.98
Qx-2	748.81	805.10	910.46	1.36	-5.54	-3.86
Y6	732.97	818.47	931.91	1.33	-5.65	-4.05

Supplementary Table. 3 Stoke's shift of Qx-1, Qx-2 and Y6 in solution and neat films.

	Solution			Film		
System	Y6	Qx-1	Qx-2	Y6	Qx-1	Qx-2
Stoke's shift	66	60	58	148	65	68

Supplementary Table. 4 Crystal data and structure refinement for Qx-1 and Qx-2.

Identification code	Qx-1	Qx-2
Empirical formula	C104 H112 F4 N8 O2 S4	C104 H110 F4 N8 O2 S4
CCDC number	2120380	2120381
Formula weight	1710.33	1708.31
Temperature/K	179.99(10)	180
Crystal system	monoclinic	monoclinic
Space group	P 2/n (13)	C 2/c
a/Å	24.6430(9)	22.9289(12)
b/Å	13.8507(2)	27.7156(9)
c/Å	28.8718(6)	17.7637(5)
α/°	90	90
β/°	103.942(3)	112.837(4)
γ/°	90	90
Volume/Å^3	9564.3(4)	10403.7(8)
Z	4	4
ρcalcg/cm^3	1.271	1.292
μ/mm^{-1}	0.245	0.355
F(000)	3864	4236.9
Crystal size/mm^3	0.63*0.10*0.02	0.65*0.04*0.03
Radiation	MoKα (λ=0.71073)	MoKα (λ=0.71073)

2Θ range for data collection/°	2.3570 to 29.0340	2.358 to 25.027
Index ranges	-29 ≤ h ≤ 29,<br>-16 ≤ k ≤ 16,<br>-34 ≤ l ≤ 34	-27 ≤ h ≤ 27,<br>-32 ≤ k ≤ 32,<br>-21 ≤ l ≤ 21
Reflections collected	22321	47779
Independent reflections	16910	9205
Goodness-of-fit on F^2	1.068	1.088
Final R indexes [I>=2σ (I)]	R1 = 0.0959,<br>wR2 = 0.2333	22.9289(12)R1 = 0.0813,<br>wR2 = 0.2193
Final R indexes [all data]	R1 = 0.1246<br>wR2 = 0.2184	R1 =0.1099<br>wR2 =0.2014

Supplementary Table. 5 Changes in the molecular state of the ground state (S_0), the lowest singlet excited state (S_1) and the ionic state during the photo-electric conversions.

Photo-electric conversions	Electronic process	Molecular state (D)	Molecualr state (A)
Exciton generation	$DA \rightarrow DA^*$	/	S_0 (A) $\rightarrow$ S_1(A)
	or $DA \rightarrow D^*A$	S_0 (D) $\rightarrow$ S_1(D)	/
Exciton diffusion	$D^*A \rightarrow DA^*$	S_1 (D) $\rightarrow$ S_0(D)	S_0 (A) $\rightarrow$ S_1(A)
	or $DA^* \rightarrow D^*A$	S_0 (D) $\rightarrow$ S_1(D)	S_1 (A) $\rightarrow$ S_0(A)
Exciton dissociation	$DA^* \rightarrow D^+A^-$	S_0(D) $\rightarrow$ cation(D)	S_1 (A) $\rightarrow$ anion (A)
	or $D^*A \rightarrow D^+A^-$	S_1(D) $\rightarrow$ cation(D)	S_0 (A) $\rightarrow$ anion (A)
Charge transport	$D^+ \leftrightarrow D$	cation(D) $\leftrightarrow$ S_0(D)	/
	$A^- \leftrightarrow A$	/	anion (A) $\leftrightarrow$ S_0 (A)
Charge recombination	$D^+A^- \rightarrow DA$	cation(D) $\rightarrow$ S_0(D)	anion (A) $\rightarrow$ S_0 (A)

Supplementary Table. 6 Device optimization for PM6:Qx-1 blends.

D/A	additives	Thermal annealing (°C)	V_{OC}(V)	J_{SC} (mA cm^{-2})	FF(%)	PCE(%)
1:1.2	0.5%CN	100 (10min)	0.915	24.44	73.49	16.43
1:1.3	0.5%CN	100 (10min)	0.916	24.54	73.41	16.50
1:1.4	0.5%CN	100 (10min)	0.915	25.00	74.40	17.02
1:1.5	0.5%CN	100 (10min)	0.915	25.59	73.29	17.17
1:1.6	0.5%CN	100 (10min)	0.911	24.73	75.39	16.99
1:1.5	0.5%CN	100 (10min)	0.915	25.59	73.29	17.17
1:1.5	0.6%CN	100 (10min)	0.911	26.09	75.46	17.94
1:1.5	0.7%CN	100 (10min)	0.919	25.08	76.55	17.63
1:1.5	0.6%CN	90 (10min)	0.920	24.85	75.43	17.23
1:1.5	0.6%CN	120 (10min)	0.910	22.51	71.01	14.54
1:1.5	0.6%CN	140 (10min)	0.923	21.04	69.62	13.52
1:1.5	0.6%CN	160 (10min)	0.928	19.63	69.68	12.70
1:1.5	0.6%CN	180 (10min)	0.921	18.93	66.67	11.62

Supplementary Table. 7 Device optimization for PM6:Qx-2 blends**.**

D/A	additive	Thermal annealing (°C)	V_{OC} (V)	J_{SC} (mA cm^{-2})	FF(%)	PCE(%)
1:1.1	0.5%CN	110 (10min)	0.941	24.30	72.94	16.67
1:1.2	0.5%CN	110 (10min)	0.936	25.35	73.91	17.53
1:1.3	0.5%CN	110 (10min)	0.938	25.43	75.17	17.92
1:1.4	0.5%CN	110 (10min)	0.942	24.65	72.58	16.85
1:1.3	0.4%CN	110 (10min)	0.949	25.34	70.55	16.97
1:1.3	0.55%CN	110 (10min)	0.934	26.47	73.69	18.22
1:1.3	0.6%CN	110 (10min)	0.934	26.53	73.73	18.22
1:1.3	0.7%CN	110 (10min)	0.938	25.00	74.69	17.52
1:1.3	0.6%CN	100 (10min)	0.938	23.63	74.41	16.49
1:1.3	0.6%CN	120 (10min)	0.934	25.44	73.65	17.51
1:1.3	0.6%CN	140 (10min)	0.926	23.85	72.62	16.03
1:1.3	0.6%CN	160 (10min)	0.931	23.96	71.05	15.85
1:1.3	0.6%CN	180 (10min)	0.903	23.24	68.12	14.30

Supplementary Table. 8 Device parameters for high performance binary OSCs in the literature.

Acceptor	Donor	V_{OC} (V)	PCE (%)	FF	J_{SC} (mA cm^{-2})	Energy loss (eV)	Ref. (SI)
AQx2	PM6	0.86	16.64	0.76	25.4	0.54	2
BTP-4Cl	PM6	0.87	16.5	0.75	25.4	0.533	25
BTP-4F-12	PM6	0.86	16.2	0.76	25.3	0.539	26
BTP-Ec9	PM6	0.84	17.8	0.81	26.2	0.561	27
BTP-Ec11	PM6	0.85	16.9	0.77	25.7	0.549	27
Y18	PM6	0.84	16.52	0.76	25.7	0.53	28
BTIC-2Cl-yCF3	PM6	0.84	16.31	0.77	25.1	0.60	29
L8-BO	PM6	0.87	18.32	0.82	25.72	0.55	18
Y6	PM6	0.84	16.61	0.76	25.91	0.56	18
L8-HD	PM6	0.88	17.39	0.79	25.08	0.55	18
L8-OD	PM6	0.89	16.26	0.75	24.57	0.53	18
BTP-4F-P2EH	PM6	0.88	18.22	0.80	25.85	0.57	30
BTP-C9-N4F	PM6	0.85	17.0	0.76	26.3	0.54	31
L8-BO-F	PM6	0.934	16.82	0.77	23.42	0.513	32
BTP-S2	PM6	0.945	16.37	0.72	24.07	0.53	33
Y11	PM6	0.853	16.50	0.731	26.56	0.51	34
Y11	PM6	0.833	16.54	0.7433	26.74	0.49	34

Supplementary Table. 9 Device parameters for high performance ternary OSCs in the literature.

Material-1	Material-2	Material-3	V_{OC}(V)	PCE(%)	FF	J_{SC} (mA cm^{-2})	Energy loss (eV)	Ref. (SI)
BTIC-2Cl-yCF3	PM6	PC71ThBM	0.85	17.12	0.78	25.8	0.56	[29]
BTP-eC9	PM6	PM6-Si30	0.870	18.27	0.78	26.22	0.51	[35]
BTP-4F-12	PM6	MeIC	0.863	17.4	0.79	25.4	0.526	[26]
BTP-eC9	PM6	HDO-4Cl	0.866	18.86	0.81	25.4	0.53	[36]
eC9-2Cl	PBQx-TF	F-BTA3	0.879	19.0	0.809	26.4	0.551	[37]
Y6	PM6	TiC12	0.853	17.25	0.754	26.80	0.55	[38]
BTP-eC9	PM6	L8-BO-F	0.853	18.66	0.8	27.35	0.519	[32]
Y6	PM6	DRTB-T-C4	0.854	17.05	0.81	24.68	0.559	[39]
Y6	PM6	S3	0.856	17.53	0.79	25.86	0.579	[40]
Y6	PM6	ZY-4Cl	0.88	17.6	0.75	25.7	0.538	[41]

Supplementary Table. 10 Photovoltaic parameters of three devices based on PM6:Qx-2 measured both in our lab and in Prof. Zhishan Bo's lab.

Number	condition	V_{OC} (V)	J_{SC} (mA/ cm^2)	FF (%)	PCE (%)
No. 1	Our lab	0.933	25.53	76.13	18.13
	Prof. Bo's lab	0.930	25.64	75.77	18.07
No. 2	Our lab	0.936	26.09	74.69	18.23
	Prof. Bo's lab	0.934	25.81	74.43	17.94
No. 3	Our lab	0.927	25.99	75.46	18.19
	Prof. Bo's lab	0.927	25.81	75.02	17.94

Supplementary Table. 11 The hole mobility and electron mobility.

Films	μ_e (cm^2 V^{-1} s^{-1})	μ_h (cm^2 V^{-1} s^{-1})	μ_h/μ_e
PM6:Qx-1	1.88×10^{-4}	3.64×10^{-4}	1.94
PM6:Qx-2	2.13×10^{-4}	3.39×10^{-4}	1.59
PM6:Y6	1.33×10^{-4}	3.36×10^{-4}	2.53
Qx-1	4.56×10^{-4}	-	-
Qx-2	6.98×10^{-4}	-	-
Y6	3.42×10^{-4}	-	-

Supplementary Table. 12 The quantum yield (QY) of Qx-1, Qx-2 and Y6.

Materials	Qx-1	Qx-2	Y6
QY (800-950nm）	7.65%	12.34%	6.42%

Supplementary Table. 13 The related parameters of decay dynamics excitons.

Samples	t_0 [ps]	k [s^{-1} × 10^9]	γ [cm^3 ps^{-1} × 10^{-9}]	D [cm^2 s^{-1}× 10^{-3}]	L [nm]
Y6	896.2	1.12	3.12	1.24	10.6
Qx-1	878.8	1.14	2.58	1.03	9.5
Qx-2	1089.0	0.91	3.65	1.45	12.6

Supplementary References

1. Song, H., Lee, T., Han, M., Lee, J. & Moon, D. Synthesis of Donor-Acceptor polymers through control of the chemical structure: Improvement of PCE by planar structure of polymer backbones. *Polymer* **54**, 1072-1079 (2013).

2. Zhou, Z. et al. Subtle Molecular Tailoring Induces Significant Morphology Optimization Enabling over 16% Efficiency Organic Solar Cells with Efficient Charge Generation. *Adv. Mater.* **32**, 1906324 (2020).

3. Mo, D. et al. Alkyl chain engineering of chlorinated acceptors for elevated solar conversion. *J. Mater. Chem. A* **8**, 8903-8912 (2020).

4. Sun, Q., Wang, H., Yang, C. & Li, Y. Synthesis and electroluminescence of novel copolymers containing crown ether spacers. *J. Mater. Chem.* **13**, 800-806 (2003).

5. Bin, H. et al. 11.4% Efficiency non-fullerene polymer solar cells with trialkylsilyl substituted 2D-conjugated polymer as donor. *Nat. Commun.* **7**, 13651 (2016).

6. Stein, T., Eisenberg, H., Kronik, L. & Baer, R. Fundamental gaps in finite systems from eigenvalues of a generalized Kohn-Sham method. *Phys. Rev. Lett.* **105**, 266802 (2010).

7. Pandey, L., Doiron, C., Sears, J. & Bredas, J. Lowest excited states and optical absorption spectra of donor-acceptor copolymers for organic photovoltaics: a new picture emerging from tuned long-range corrected density functionals. *Phys. Chem. Chem. Phys.* **14**, 14243-14248 (2012).

8. Shen, X., Han, G. & Yi, Y. The nature of excited states in dipolar donor/fullerene complexes for organic solar cells: evolution with the donor stack size. *Phys. Chem. Chem. Phys.* **18**, 15955-15963 (2016).

9. Shuai, Z. Thermal Vibration Correlation Function Formalism for Molecular Excited State Decay Rates. *Chin. J. Chem.* **38**, 1223-1232 (2020).

10. Frisch, M. et al. Gaussian 09 Rev. D.01. (2016).

11. Hess, B., Kutzner, C., van der Spoel, D. & Lindahl, E. GROMACS 4: Algorithms for Highly Efficient, Load-Balanced, and Scalable Molecular Simulation. *J. Chem. Theory Comput.* **4**, 435-447 (2008).

12. Koster, L., Kemerink, M., Wienk, M., Maturova, K. & Janssen, R. Quantifying bimolecular recombination losses in organic bulk heterojunction solar cells. *Adv. Mater.* **23**, 1670-1674 (2011).

13. Kyaw, A. et al. Intensity dependence of current-voltage characteristics and recombination in high-efficiency solution-processed small-molecule solar cells. *ACS Nano* **7**, 4569-4577 (2013).

14. Li, G. et al. Efficient modulation of end groups for the asymmetric small molecule acceptors enabling organic solar cells with over 15% efficiency. *J. Mater. Chem. A* **8**, 5927-5935 (2020).

15. Blom, P., deJong, M. & Vleggaar, J. Electron and hole transport in poly(p-phenylene vinylene) devices. *Appl. Phys. Lett.* **68**, 3308-3310 (1996).

16. Chandrabose, S. et al. High Exciton Diffusion Coefficients in Fused Ring Electron Acceptor Films. *J. Am. Chem. Soc.* **141**, 6922-6929 (2019).

17. Zhang, G. et al. Delocalization of exciton and electron wavefunction in non-fullerene acceptor molecules enables

efficient organic solar cells. *Nat. Commun.* **11**, 3943 (2020).

18. Li, C. et al. Non-fullerene acceptors with branched side chains and improved molecular packing to exceed 18% efficiency in organic solar cells. *Nat. Energy* **6**, 605-613 (2021).

19. Yao, J. et al. Quantifying Losses in Open-Circuit Voltage in Solution-Processable Solar Cells. *Phys. Rev. Appl.* **4**, 014020 (2015).

20. Wang, Y. et al. Optical Gaps of Organic Solar Cells as a Reference for Comparing Voltage Losses. *Adv. Energy Mater.* **8**, 10 (2018).

21. Fu, H., Wang, Z. & Sun, Y. Polymer Donors for High-Performance Non-Fullerene Organic Solar Cells. *Angew. Chem. Int. Ed.* **58**, 4442-4453 (2019).

22. Liu, T. et al. Optimized Fibril Network Morphology by Precise Side-Chain Engineering to Achieve High-Performance Bulk-Heterojunction Organic Solar Cells. *Adv. Mater.* **30**, e1707353 (2018).

23. Classen, A. et al. The role of exciton lifetime for charge generation in organic solar cells at negligible energy-level offsets. *Nat. Energy* **5**, 711-719 (2020).

24. Firdaus, Y. et al. Long-range exciton diffusion in molecular non-fullerene acceptors. *Nat. Commun.* **11**, 5220 (2020).

25. Cui, Y. et al. Over 16% efficiency organic photovoltaic cells enabled by a chlorinated acceptor with increased open-circuit voltages. *Nat. Commun.* **10**, 2515 (2019).

26. Ma, X. et al. Achieving 17.4% Efficiency of Ternary Organic Photovoltaics with Two Well-Compatible Nonfullerene Acceptors for Minimizing Energy Loss. *Adv. Energy Mater.* **10**, 2001404 (2020).

27. Cui, Y. et al. Single-Junction Organic Photovoltaic Cells with Approaching 18% Efficiency. *Adv. Mater.* **32**, 1908205 (2020).

28. Zhu, C. et al. Tuning the electron-deficient core of a non-fullerene acceptor to achieve over 17% efficiency in a single-junction organic solar cell. *Energy Environ. Sci.* **13**, 2459-2466 (2020).

29. Chen, H. et al. 17.1 %-Efficient Eco-Compatible Organic Solar Cells from a Dissymmetric 3D Network Acceptor. *Angew. Chem. Int. Ed.* **60**, 3238-3246 (2021).

30. Zhang, J. et al. Alkyl-Chain Branching of Non-Fullerene Acceptors Flanking Conjugated Side Groups toward Highly Efficient Organic Solar Cells. *Adv. Energy Mater.* **11**, 2102596 (2021).

31. Shi, Y. et al. Optimizing the Charge Carrier and Light Management of Nonfullerene Acceptors for Efficient Organic Solar Cells with Small Nonradiative Energy Losses. *Sol. RRL* **5**, 2100008 (2021).

32. Cai, Y. et al. A Well-Mixed Phase Formed by Two Compatible Non-Fullerene Acceptors Enables Ternary Organic Solar Cells with Efficiency over 18.6%. *Adv. Mater.* **33**, 2101733 (2021).

33. Li, S. et al. Asymmetric Electron Acceptors for High-Efficiency and Low-Energy-Loss Organic Photovoltaics. *Adv. Mater.* **32**, 2001160 (2020).

34. Liu, S. et al. High-efficiency organic solar cells with low non-radiative recombination loss and low energetic disorder. *Nat. Photon.* **14**, 300-305 (2020).

35. Peng, W. et al. Using Two Compatible Donor Polymers Boosts the Efficiency of Ternary Organic Solar Cells to 17.7%. *Chem. Mater.* **33**, 7254-7262 (2021).

36. Bi, P. et al. Reduced non-radiative charge recombination enables organic photovoltaic cell approaching 19% efficiency. *Joule* **5**, 2408-2419 (2021).

37. Cui, Y. et al. Single-Junction Organic Photovoltaic Cell with 19% Efficiency. *Adv. Mater.* **33**, 2102420 (2021).

38. Tang, W. et al. 17.25% high efficiency ternary solar cells with increased open-circuit voltage using a high HOMO level small molecule guest donor in a PM6:Y6 blend. *J. Mater. Chem. A* **9**, 20493-20501 (2021).

39. Zeng, Y. et al. Exploring the Charge Dynamics and Energy Loss in Ternary Organic Solar Cells with a Fill Factor Exceeding 80%. *Adv. Energy Mater.* **11**, 2101338 (2021).

40. An, Q. et al. Two compatible polymer donors contribute synergistically for ternary organic solar cells with 17.53% efficiency. *Energy Environ. Sci.* **13**, 5039-5047 (2020).

41. Jiang, B. et al. Realizing Stable High-Performance and Low-Energy-Loss Ternary Photovoltaics through Judicious Selection of the Third Component. *Sol. RRL* **5**, 2100450 (2021).
