# Supplementary material for: Small reorganization energy acceptors enable low energy losses in non-fullerene organic solar cells
Source: Nat Commun. 2022 Jun 7;13:3256. doi: 10.1038/s41467-022-30927-y (PMC9174259; doi:10.1038/s41467-022-30927-y)
Supplement: Supplementary file 3 — Solar Cell Reporting Summary [file 41467_2022_30927_MOESM3_ESM.pdf]

Corresponding author(s): Kun Lu, Xiaotao Hao, Lingyun Zhu, Zhixiang Wei

Solar Cells Reporting Summary

Nature Research wishes to improve the reproducibility of the work that we publish. This form is intended for publication with all accepted papers reporting the characterization of photovoltaic devices and provides structure for consistency and transparency in reporting. Some list items might not apply to an individual manuscript, but all fields must be completed for clarity.

For further information on Nature Research policies, including our data availability policy, see Authors & Referees.

Experimental design

Please check: are the following details reported in the manuscript?

1. Dimensions

Item	Yes/No	Details
Area of the tested solar cells	[x] Yes [ ] No	2 x 2 mm.
Method used to determine the device area	[x] Yes [ ] No	Area of the tested solar cells is 0.04 mm^2 defined by optical microscope.

2. Current-voltage characterization

Item	Yes/No	Details
Current density-voltage (J-V) plots in both forward and backward direction	[ ] Yes [x] No	We only scan the device in forward direction. Generally no difference between these two direction for organic solar cells.
Voltage scan conditions<br>*For instance: scan direction, speed, dwell times*	[x] Yes [ ] No	Forward direction, the scan voltage from -1 to 1 V with a voltage step of 10 mV and delay time of 1ms. And all of these information has been included in method section.
Test environment<br>*For instance: characterization temperature, in air or in glove box*	[x] Yes [ ] No	Our devices were characterized at room temperature (ca. 25 Celsius degree) in glove box.
Protocol for preconditioning of the device before its characterization	[ ] Yes [x] No	No preconditioning protocol.
Stability of the J-V characteristic<br>*Verified with time evolution of the maximum power point or with the photocurrent at maximum power point; see ref. 7 for details.*	[ ] Yes [x] No	Organic photovoltaic devices show no decay or instability during the test of J-V characteristics.

3. Hysteresis or any other unusual behaviour

Item	Yes/No	Details
Description of the unusual behaviour observed during the characterization	[ ] Yes [x] No	No hysteresis or other unusual behaviour was observed during the characterization of the solar cells. In general, organic solar cells do not have hysteresis problems.
Related experimental data	[ ] Yes [x] No	No hysteresis or other unusual behaviour was observed during the characterization of the solar cells.

4. Efficiency

Item	Yes/No	Details
External quantum efficiency (EQE) or incident photons to current efficiency (IPCE)	[x] Yes [ ] No	EQE is provided using Oriel Newport system (Model 66902) equipped with a standard Si diode in air condition.
A comparison between the integrated response under the standard reference spectrum and the response measure under the simulator	[x] Yes [ ] No	The difference between the integrated current from EQE and the short-circuit current from J-V curve measured under AM 1.5G solar simulator is within 5% difference which is within the accuracy confidence of the measurements.
For tandem solar cells, the bias illumination and bias voltage used for each subcell	[ ] Yes [x] No	We did not fabricate the tandem solar cells in this work.

5. Calibration

Item	Yes/No	Details
Light source and reference cell or sensor used for the characterization	[x] Yes [ ] No	J–V curves measurements were conducted under AM 1.5 G (100mWcm−2) by Newport Thermal Oriel 91159A solar simulator, with Newport Oriel PN 91150 V Si-based solar cell as light intensity calibration reference.
Confirmation that the reference cell was calibrated and certified	[x] Yes [ ] No	The reference cell was calibrated and certified.

Calculation of spectral mismatch between the reference cell and the devices under test

☒ Yes ☐ No

Less than 3%.

6. Mask/aperture

Size of the mask/aperture used during testing

☐ Yes ☒ No

No, we didn't use mask in this measurement. According to our previous experience, when we use mask, the Voc and Jsc are a little lower, the FF is higher, the power conversion efficiency is equivalent compared with no mask, the variation is within 0.5%.

Variation of the measured short-circuit current density with the mask/aperture area

☐ Yes ☒ No

No, we didn't use mask in this measurement.

7. Performance certification

Identity of the independent certification laboratory that confirmed the photovoltaic performance

☒ Yes ☐ No

We have given Certification report by National Institute of Metrology (NIM), China. in Supplementary Figure 11 .

A copy of any certificate(s)
Provide in Supplementary Information

☒ Yes ☐ No

We have given Certification report by National Institute of Metrology (NIM), China. in Supplementary Figure 11 .

8. Statistics

Number of solar cells tested

☒ Yes ☐ No

The average PCE of the OSCs is obtained from 30 independent devices.

Statistical analysis of the device performance

☒ Yes ☐ No

We have given statistical data of device performance in Table 1.

9. Long-term stability analysis

Type of analysis, bias conditions and environmental conditions
For instance: illumination type, temperature, atmosphere humidity, encapsulation method, preconditioning temperature

☒ Yes ☐ No

The stability of these devices were evaluated in glove box by repeating to test the J-V curves during the about 1000 storage hours at room temperature under unencapsulated condition, and the results can be seen in Supplementary Figure 13 .
